# Supplementary material for: From reads to operational taxonomic units: an ensemble processing pipeline for MiSeq amplicon sequencing data
Source: Gigascience. 2017 Jan 18;6(2):1–10. doi: 10.1093/gigascience/giw017 (PMC5466709; doi:10.1093/gigascience/giw017)
Supplement: GIGA-D-16-00030_Revision_1.pdf [file giw017_GIGA-D-16-00030_Revision_1.pdf]

Manuscript

Click here to view linked References

Click here to download Manuscript Manuscript_GigaScience_Track_Changes.docx

From reads to operational taxonomic units: an ensemble processing pipeline for MiSeq amplicon sequencing data

Mohamed Mysara[1,2,3,4], Mercy Njima[1], Natalie Leys[1], Jeroen Raes[2,3,4] and Pieter Monsieurs[1,*]

[1]Unit of Microbiology, Belgian Nuclear Research Centre (SCK-CEN), 2400, Mol, Belgium.

[2]Department of Bioscience Engineering, Vrije Universiteit Brussel, Brussels, Belgium.

[3]VIB Center for the Biology of Disease, VIB, Leuven, Belgium.

[4]Department of Microbiology and Immunology, REGA institute, KU Leuven, Belgium.

Correspondence to P.M. email: pieter.monsieurs@sckcen.be.

Abstract

Introduction: The development of high-throughput sequencing technologies has provided microbial ecologists with an efficient approach to assess bacterial diversity at an unseen depth, particularly with the recent advances in the Illumina MiSeq sequencing platform. However, analysing such high-throughput data is posing important computational challenges, requiring specialized bioinformatics solutions at different stages during the processing pipeline, such as assembly of paired-end reads, chimera removal, correction of sequencing errors and clustering of those sequences into Operational Taxonomic Units (OTUs). Individual algorithms grappling with each of those challenges have been combined into various bioinformatics pipelines, such as mothur, QIIME, LotuS and USEARCH.

Results: Using a set of well-described bacterial mock communities, state-of-the-art pipelines for Illumina MiSeq amplicon sequencing data are benchmarked at the level of the amount of sequences retained, computational cost, error rate and quality of the OTUs. In addition, a new pipeline called OCToPUS is introduced, which is making an optimal combination of different algorithms. Huge variability is observed between the different pipelines in respect to the monitored performance parameters, where in general the amount of retained reads is found to be inversely proportional to the quality of the reads. By contrast, OCToPUS achieves the lowest error rate, minimum number of spurious OTUs, and the closest correspondence to the existing community, while retaining the uppermost amount of reads when compared to other pipelines.

Conclusion: The newly introduced pipeline translates Illumina MiSeq amplicon sequencing data into high-quality and reliable OTUs, with improved performance and accuracy compared to the currently existing pipelines.

Keywords

16S rRNA metagenomics, amplicon sequencing, chimera, denoising, OTU clustering, operational taxonomic units

Background

The application of new high-throughput sequencing technologies to assess microbial diversity is a fast-evolving discipline. The high-throughput capacity of those technologies and the absence of the need to culture and isolate microbial species provides researchers in the field with a very powerful technology. The sequencing of the 16S rRNA gene as phylogenetic marker gene is very often used approach to assess the microbial diversity. The short length of the reads currently produced by most sequencing technologies is an important limitation, as those reads only cover one or a few variable regions within the 16S rRNA gene. However, this drawback is largely compensated by the huge reduction in economic cost and increase in throughput compared to traditional approaches.

The Roche 454 pyrosequencing technology was the first high-throughput sequencing technology to be used in microbial ecology studies [1,2], followed by other technologies such as Ion Torrent [3], and Illumina [4] and PacBio [5]. The introduction of the Illumina MiSeq platform, offering paired-end reads nowadays up to 2 x 300 bp at a reasonably high throughput combined with the announcement of Roche to shut down its 454 sequencing services by 2016, led to a shift towards the former technology. Therefore, results presented within this work are focused on sequencing data obtained from the Illumina MiSeq platform.

The ultimate goal of these amplicon sequencing approaches is to obtain a holistic view on the microbial composition within a sample, mostly obtained via binning the sequencing reads based on their sequence similarity to each other, resulting in clusters of reads, commonly referred to as Operational Taxonomic Units (OTUs). Eventually, in the ideal scenario each OTU should represent an actual bacterial species. Nonetheless, many researchers have reported an inflation of the number of OTUs when sequencing mock communities. Such an approach of using a well-defined mixture of microbial cells allows gaining insight into the numerous sources of errors potentially hampering the correct interpretation of amplicon sequencing data [6–8]. A first source of errors originates from chimera formation within the PCR amplification step, thereby creating a chimeric sequence which consists of two or more fragments from distinct species [9–12] As those chimeras will propagate in the same way as any other DNA sequence, they can take up to 30% of all unique sequencing reads. Falling short in removal of these artificial sequences will have a huge impact on the diversity estimates, since chimeras that go undetected will be interpreted as novel species [13,14]. Secondly, the high-throughput character of the new sequencing platforms comes at the cost of a decreased accuracy, in such a way posing important challenges at the level of data analysis. Illumina sequencing platforms suffer mainly from substitutions-type miscalls which frequently accompanies GC-rich regions [15–17] or which are caused by improper phasing/prephasing [18], or which resulted from the high correlation of emission spectra between A and C as well as G and T [18–20]. Additionally, to obtain reads with an acceptably low error rate, both forward and reverse reads needed to be at least partially overlapping,

thus allowing the combination of the prediction in both reads to generate a consensus amplicon [6]. Yet, as this overlapping region spans those parts of the reads with the lowest quality scores, such practice can still introduce errors, especially when conflicts between both reads occur.

Numerous bioinformatics algorithms have been developed for the different steps within the workflow of amplicon sequencing data produced by the Illumina MiSeq platform, such as: a) paired-end assembly, by merging both forward and reverse reads into one consensus sequence, b) quality filtering, via filtering reads with low sequencing quality, c) denoising i.e. correction of sequencing errors, d) the removal of chimeric reads and e) clustering via binning the sequencing reads into OTUs based on their sequence similarity to each other. An overview of previously developed algorithms is given in table 1. Integration of those single-step tools into pipelines covering the whole processing stage, resulted in different workflows including MG-RAST [21], mothur [22], QIIME [23], USEARCH [24], LotuS [25], and BioMaS [26].

Various efforts have been made to compare the different individual tools developed for each preprocessing step, e.g. there exist benchmark studies comparing the paired-end assemblers [7,8], denoising tools [6–8,27,28], chimera detection tools [29,30] and clustering algorithms [31–34]. However, limited literature is available comparing pipelines, such as USEARCH, LotuS, mothur, and QIIME. Such a benchmarking analysis could provide crucial information to microbial ecologists in terms of accuracy, computational time, and retained sample size, as such offering guidance towards the selection of the appropriate pipeline. First initiatives to perform such benchmark have already led to interesting results (Plummer et al. [35], Hildebrand et al. [25], Fosso et al. [26], D'Argenio et al. [36]). However, each of those comparative studies used either biological samples or simulated datasets, thus making it difficult to assess the quality in terms of error rate and OTU accuracy.

In this work, a comprehensive comparison was made between mothur, QIIME, LotuS and USEARCH pipelines, in respect to reads throughput, error rate and OTU accuracy. We also propose within this work a novel pipeline that combines the advantages of different existing individual tools, which is entitled OCToPUS (**O**ptimized **C**A**T**Ch, m**o**thur, I**P**ED, **U**PARSE and **S**PAdes). In contrast to previous comparative analyses described above, we used mock community datasets, as such providing a benchmark that can be used to calculate the error rate and correspondence of the resulting OTUs with the actual microbial composition. Important to notice is that this work has no intention of comparing the underlying individual algorithms built-in within each pipeline. It rather treats the entire pipeline as a black box and assesses the accuracy using a unified evaluation process apart from the implemented individual algorithms.

Data Description

The benchmark analysis between various pipelines for 16S rRNA amplicon sequencing was done using mock samples with a known microbial composition. Unlike the use of simulated data or real biological samples, this type of samples allows for an accurate assessment of the error rates and microbial compositions returned by each pipeline. Thus, thirteen publicly available Illumina MiSeq sequencing samples were used representing three different mock communities. The first mock community – called MOCK1 – contains of 21 species, and the corresponding amplicon sequencing data set covers the V34 and V4 regions of the 16S rRNA gene, each amplicon sequenced in triplicate (run IDs 130403, 130417 and 130422). The second mock community (MOCK2) consists of 20 different organisms covering the V4 and V45 regions, each of them sequenced in duplicate (named V4.I.1 and V4.I.05 (for V4), V4.V5.I.1 and V4.V5.I.11 (for V45). The third mock community (MOCK3) consists of 12 species, is sequenced in triplicate (named M1, M2, M3) and covers the V34 region. MOCK1 is available via (http://www.mothur.org/MiSeqDevelopmentData.html) under accession 130403, 130417 and 130422, MOCK2 is available via European Bioinformatics Institute Nucleotide Archive SRA under project ID PRJEB4688 and MOCK3 is available via National Center for Biotechnology Information SRA under project ID: SRP066114. The detailed composition, library preparation and sequencing on the Illumina MiSeq platform are described in detail in Supplementary File 1 as well as the respective publications for MOCK1 [6], MOCK2 [37], MOCK3 [7].

Methods

Standardization of the Pipelines

The samples were analysed using four pipelines: QIIME (Version 1.8.0), mothur (Version 1.33.3), LotuS (Version 1.506), USEARCH (Version v8.1.1861_i86linux32) and a new pipeline OCToPUS introduced within this work. In general, the standard commands were used for each pipeline, i.e. using the default parameters. However, in order to allow for a fair comparison on the number of spurious OTUs, OTUs were not rejected based on their relative abundance or their taxonomic classifications in any of the pipelines. This necessitated the deactivation of default singleton removal option in UPARSE and skipping the default *remove.lineage* step in mothur or putting the *keepUnclassified* parameter in LotuS. For the same reason the *reference* based mode of the chimera detection for all pipelines was not included. A detailed description of the commands used within each pipeline is described below, and a schematic overview of the different steps is summarized in Figure 1.

Mothur

In general, the Standard Operation Procedure of mothur for analysing 16S rRNA amplicon sequencing data (http://www.mothur.org/wiki/MiSeq_SOP, d.d. 2015-11-23) is used as guideline. In a first step, the forward and reverse reads are merged using the *make.contigs* command. Based on the quality

scores, a heuristic has been implemented to resolve conflicts between both reads, thereby replacing problematic conflicts with "N". Reads exhibiting any ambiguous positions or containing a more than 8-base homopolymer are subsequently removed using the *screen.seqs* command. Next, reads are aligned to the SILVA reference database [38] using the *align.seqs* command. Those reads that fail to align to the correct location within the 16S rRNA gene [39–41] are culled using the *screen.seqs* command. Aligned reads are simplified (via removing non-informative columns (using the *filter.seqs command*), dereplicated (via the *unique.seqs* command), and denoised with mothur implementation of the Single Linkage Preclustering algorithm [42] via, the *pre.cluster* command. The resulting reads are screened for presence of chimeras using UCHIME [43] via the *chimera.uchime* command. Finally, sequences are clustered into OTUs using the *cluster.split* command.

USEARCH

Following the recommendations by Edgar and Flyvbjerg [8] and the online published USEARCH workflow (http://drive5.com/usearch/manual/uparse_pipeline.html), both forward and reverse reads are merged by aligning them using the *fastq_mergepairs* command. The *fastq_filter* command is used to assess the expected number of errors, as described in [8], and filter the reads accordingly. Dereplication is performed via the *derep_fulllength* command, followed by denoising via *cluster_fast* which is the implementation of the UNOISE algorithm [8]. Via the *sortbysize* command reads are arranged in descending order of abundance, followed by the *cluster_otus* command that combines both the OTU clustering and chimera (*de novo*) removal step. Reads are mapped to the final OTUs list using *usearch_global* command to assign abundances to each OTU and formulate the OTU-table.

QIIME

Following the recommendations on QIIME website (http://qiime.org/), first both forward and reverse reads are merged via the *join_paired_ends.py* command, an implementation of the fastq-join approach (Erik Aronesty, 2011). Next a quality filtering step based on the Phred scores is applied, as described in Bokulich et al [44] via *split_libraries_fastq.py*. Chimeras are identified using *identify_chimeric_seqs.py* command (using the usearch61 option that runs the UCHIME algorithm), and subsequently removed via *filter_fasta.py*. OTU clustering is performed using the *pick_open_reference_otus.py* command utilizing the default UCLUST algorithm and Greengenes as reference database.

LotuS

LotuS requires specifying all parameters in a single command, which is different from the step-wise approach of previous pipelines. First, LotuS reads the mapping file specifying the input fastq files, which are subsequently demultiplexed and quality filtered using the simple demultiplexer (sdm) algorithm [25]. Reads are trimmed into "seeds" with a length of 170 bases, which are clustered and checked for chimera using the UPARSE algorithm to formulate the OTU table. Next, the seed sequences of the shortlisted OTUs are extended and assembled via the sdm and Flash [45] algorithms, respectively, of which the output are used as the representative sequences of the OTUs.

OCToPUS

Within this work a new pipeline was developed which utilizes the benefits of various tools and state-of-the-art algorithms, described as an **O**ptimized **C**A**T**Ch, m**o**thur, I**P**ED, **U**PARSE and **S**PAdes, abbreviated as OCToPUS. First, both forward and reverse reads are quality checked via looking at k-mer frequency to identify potential false k-mers using the Hammer algorithm [46] implemented in the SPAdes tool [47]. Next, reads are assembled via the mothur *make.contigs* command, followed by screening, aligning, filtering and dereplication, similar to what was described in the mothur approach. Next, reads are denoised using the IPED algorithm, which applies an artificial intelligent classifier to identify and correct positions likely to be erroneous [7]. Chimera detection is performed via the CATCh algorithm, a second layer classifier that ensembles the scores of various chimera detection tools into a more accurate classification [29]. Subsequently, we apply the UPARSE clustering approach as implemented in USEARCH, using the *cluster_otus* and *usearch_global* commands to assign an abundance level to each OTU.

Evaluation Criteria

Comparison of the different pipelines was performed using four different parameters: 1) amount of reads rejected, 2) the error rate, 3) the number of OTUs and their composition and 4) computation time. The amount of reads retained within the different pipelines was calculated via the mothur *summary.seqs* command at different stages within the workflow, i.e. after paired-end assembly, after quality filtering and after chimera removal. Due to different order of the processing steps in LotuS as illustrated in Figure 1, only the final amount of reads can be reported.

Secondly, as the microbial composition of the mock sample is known – and as such the reference sequence of the corresponding 16S rRNA genes – actual error rates were calculated via the mothur *seq.error* command. The error rate was calculated by taking the ratio of the number all erroneous bases (deletions, insertions and substitutions errors) over the total number of bases. Error rates were reported twice: once after the chimeric reads were accurately removed in order to have an idea on the sequencing error rate excluding the chimeric reads, and a second time after applying a regular chimera removal tool as implemented within each pipeline, thereby giving a more realistic estimation of the total error rate that will be retained within the sequencing data. As LotuS – unlike the other pipelines – does only perform the paired-end assembly step after creating the OTUs, it is not possible to calculate the error rate of assembled reads prior to clustering.

Operational taxonomic units (OTUs) were assessed in a quantitative as well as a qualitative way. For the quantitative approach, we calculated the number of OTUs produced via each pipeline per sample. Those numbers were plotted using rarefaction curves where the number of OTUs are shown in the vertical axis and read counts in the horizontal axis, reflecting the influence of sequencing depth on the number of OTUs. Additionally, we performed a qualitative analysis, following a similar approach as described in Edgar [33], where the OTUs were classified into four different categories: 1) original

(more than 97% sequence similarity to a species within the mock community), 2) chimeric (similar to two or more species within the mock community), 3) contaminant (non-intended read with high sequence identity match to a species not in the targeted community), and 4) others (not fulfilling any of previous criteria).

Lastly, the computation time was calculated for the different steps within each pipeline: paired-end assembly, quality filtering (with denoising when integrated in the pipeline), chimera removal and OTU clustering using eight Intel Xeon E5-2640 2.50 GHz CPUs. The six samples of MOCK 1 were used for this analysis (with a coverage ranging from 20,000 to 700,000 reads).

Analyses and Discussion

In this work, a novel pipeline named OCToPUS is introduced, incorporating various tools that tackle the individual challenges related to 16S rRNA amplicon sequencing data analysis, such as denoising, quality filtering, chimera detection and OTU clustering. For this purpose, we utilized the commonly used mothur software pipeline as backbone, in which we replaced some of the default programs by our own selection of tools. For the denoising step, the IPED algorithm was selected as it was able to correct double the amount of errors and significantly reduce the number of spurious OTUs compared to other algorithms available [7]. For the removal of chimeric sequences, the CATCh algorithm was implemented combining the advantages of various chimera detection tools into one ensemble algorithm, thereby incorporating all individual predictions into one combined score. As such, applying CATCh has been found to increase the sensitivity (i.e., detecting true chimera) with 8% without affecting the specificity (i.e., wrongly labelling a correct sequence as chimeric) [29]. Concerning the OTU clustering step, UPARSE has been selected, as it has been proven to outperform the other state-of-the-art algorithms, bringing the number of OTUs closer to the actual number of species [33]. Additionally, we wanted to test the idea of incorporating a pre-assembly quality filtering step. Despite the fact that this step has not yet been incorporated in most pipelines, evaluation of the end results of our analysis pipeline showed a significant beneficial effect on the error rate (5% less) and the number of spurious OTUs (9% less).

Next, a benchmark analysis was conducted between our newly introduced OCToPUS pipeline and the existing state-of-the-art pipelines QIIME, USEARCH, LotuS, and mothur. A set of performance parameters were defined to assess each pipeline, i.e. the amount of reads retained, the error rates, the computational time and the quality of the OTU clustering results. For this purpose, 13 mock samples with a known composition and originating from three different studies (six sequencing runs) were processed by all four pipelines, allowing us to calculate of the four performance parameters for each pipeline. Although each pipeline was initially exposed to the same number of reads, the amount of reads retained by each of the workflows was dramatically differing between each of them. The

percentages of rejected reads were on average 23%, 24%, 26%, 26%, and 47% for LotuS, OCToPUS, QIIME, mothur and USEARCH, respectively (see supplementary file 2). Important to notice is that the amount of reads lost within a certain step is differing dramatically between different pipelines (see Figure 2), e.g. most of the reads are thrown away by QIIME during the assembly phase, while most of the reads are rejected by USEARCH in the quality filtering step.

As the main reason for rejecting those reads was to get rid of poor quality or chimeric sequences, it was utterly important to assess their influence on the error rate obtained with each approach. In a first scenario chimeras were identified by using the known reference sequences for each community, and subsequently the error rate was calculated. It is important to notice that such an analysis can only be performed for mock communities, and is performed within this context purely as benchmark analysis. Within this context, OCToPUS obtained an error rate of 0.08% on average, while USEARCH, mothur and QIIME reduced the overall error rate to 0.14%, 0.15%, and 0.47% respectively, averaged over all mock communities (see Table 2). With the exception of OCToPUS, there was a strong correlation between the amount of rejected reads and the extent to which the error rate has been reduced. Additionally, we assessed the error rate within the second scenario, where the removal of chimeras occurs using a traditional chimera detection algorithm, as such reflecting a real-life scenario. OCToPUS was able to reduce the error rate to 0.19% while USEARCH, mothur and QIIME achieved 0.23%, 0.24%, and 0.59% respectively averaged over all mock communities (see Table 2). Due to the presence of some undetected chimeras, an inflation of the error rate was reported for the second scenario compared to the first one. Nonetheless, in both scenarios the OCToPUS pipeline was deemed successful in acquiring the highest quality in respect to the error rate of the sequencing reads, without affecting the amount of reads retained. As discussed in the methods LotuS could not be included in this analysis.

The negative effect of sequencing errors and PCR artefacts are expected to influence the amount of spurious OTUs, thus a successful removal of these errors should ideally be reflected in a decrease of the number of OTUs. Although the number of OTUs are affected by the amount of reads and the level of complexity within the mock samples, [6], it has commonly been used by others as a metric for sequencing quality[6,8,27,28,30,42,48,49]. Thus, we calculated the average number of spurious OTUs –exceeding the expected number of OTUs –for all samples. OCToPUS produced on average 65 OTUs, while USEARCH, LotuS, mothur and QIIME produced 95, 208, 236, and 295 OTUs respectively (see Supplementary File 3). Using the rarefaction curves we could demonstrate that the OCToPUS pipeline was able to achieve the least amount of spurious OTUs with increasing sequencing depth, followed by USEARCH, LotuS, mothur and QIIME (see Figure 3). Nonetheless, it is important to stress that the amount of reads removed by USEARCH – the pipeline with the second best performance – is drastically lower compared with the other pipelines, as illustrated in supplementary file 2.

Achieving the least number of spurious OTUs, does not automatically imply that it will return OTU clustering results that reflect accurately the microbial composition within the mock community. Therefore, we performed an additional analysis to qualitatively assess the composition of the OTUs produced via each pipeline. Based on the classification used in Edgar et al. [33], the percentage of original species, escaped chimeras, existing contaminants and other unidentifiable sequences were calculated (see methods). Based on Figure 4, USEARCH, OCToPUS and QIIME report the most accurate correspondence to the original species, and USEARCH and OCToPUS report the least amount of chimera. The remainder of the OTUs represented contaminating reads or unidentified sequences (possibly formed via a combination of contaminants and PCR or sequencing errors). For the MOCK1 (V34) and MOCK2 (V45) samples USEARCH obtained a better prediction of the microbial community than OCToPUS. However, it is important to notice that USEARCH throws away on average 94% and 59% of the sequencing reads in MOCK1(V34) and MOCK2 (V45) samples respectively during processing – as such limiting the analysis to a small fraction of reads – while OCToPUS rejects on average 13 and 46% of the reads respectively. Similarly, LotuS throws away only 53% of the MOCK1 sequencing data (V34), yet obtaining a slightly better prediction compared to OCToPUS. Finally, we evaluated the number of species that were split over more than one OTU, and the species that were absent in the OTU production. All approaches were able to identify all species within the MOCK1 and MOCK2 communities. However, only OCToPUS and USEARCH reported an average of 1 OTU per species, while LotuS reported 1.4 OTUs per species, mothur 1.7 OTUs per species and QIIME 5.2 OTUs per species, indicating a more pronounced over-splitting effect, it was also reported with MOCK3 samples (see Supplementary File 4).

The computational cost for USEARCH and LotuS was dramatically lower compared to the other pipelines, as it only required a few seconds to process the six samples of MOCK1. Mothur, OCToPUS, and QIIME required 2.1, 2.7, and 3 minutes respectively, (see Supplementary File 5). The computational time for mothur is evenly distributed across the different steps. For OCToPUS the most time-consuming step is the paired-end assembly (including the preceding pre-assembly error correction) and chimera removal (requiring the execution of three chimera detection algorithms). Concerning QIIME, most of the computational time was dedicated to the OTU clustering step. As discussed earlier, the added computational burden for OCToPUS was overshadowed by the quality of the processed data.

Conclusively, our proposed pipeline OCToPUS combines the advantages of mothur, CATCh, IPED, UPARSE and SPAdes and was on average able to achieve the lowest error rate, the minimum number of spurious OTUs and the closest correspondence to the existing community without compromising the amount of reads retained. With the exception of USEARCH, the required computation time was in line with the other pipelines. All included algorithms are freely available – with exception of the

USEARCH licence that can be obtained from its author upon registration. Finally, our newly proposed OCToPUS pipeline is able to translate amplicon sequencing data into high-quality OTUs.

Availability and requirements

- Project name: OCToPUS
- Project home page:. https://github.com/M-Mysara/OCToPUS
- Operating system(s): UNIX
- Programming language: Perl
- Other requirements: e.g. Java 1.3.1 or higher, Perl
- License: e.g. GNU GPL (except with UPARSE, licence should be obtained directly from http://www.drive5.com/usearch/
- MOCK1 is available via (http://www.mothur.org/MiSeqDevelopmentData.html) under accession 130403, 130417 and 130422.
- MOCK2 is available via European Bioinformatics Institute Nucleotide Archive SRA under project ID PRJEB4688
- MOCK3 is available via National Center for Biotechnology Information SRA under project ID: SRP066114

Competing interests

The authors declare that they have no competing interests.

Funding

This work is funded by an SCK-CEN PhD Grant.

Authors' contributions

Conceived and designed the experiment: MM, MJ, JR and PM. Computational analysis and analysis of the data: MM, MJ and PM. Wrote the paper: MM, NL, JR and PM. All authors read and approved the final manuscript

References

1. Edwards RA, Rodriguez-Brito B, Wegley L, Haynes M, Breitbart M, Peterson DM, et al. Using pyrosequencing to shed light on deep mine microbial ecology. BMC Genomics. 2006;7:57.

2. Sogin ML, Morrison HG, Huber JA, Mark Welch D, Huse SM, Neal PR, et al. Microbial diversity in the deep sea and the underexplored "rare biosphere". Proc. Natl. Acad. Sci. U. S. A. 2006;103:12115–20.

3. Jünemann S, Prior K, Szczepanowski R, Harks I, Ehmke B, Goesmann A, et al. Bacterial community shift in treated periodontitis patients revealed by Ion Torrent 16S rRNA gene amplicon sequencing. PLoS One. 2012;7:e41606.

4. Gloor GB, Hummelen R, Macklaim JM, Dickson RJ, Fernandes AD, MacPhee R, et al. Microbiome profiling by Illumina sequencing of combinatorial sequence-tagged PCR products. PLoS One. 2010;5:e15406.

5. Fichot EB, Norman RS. Microbial phylogenetic profiling with the Pacific Biosciences sequencing platform. Microbiome. 2013;1:10.

6. Kozich JJ, Westcott SL, Baxter NT, Highlander SK, Schloss PD. Development of a dual-index sequencing strategy and curation pipeline for analyzing amplicon sequence data on the MiSeq Illumina sequencing platform. Appl. Environ. Microbiol. 2013;79:5112–20.

7. Mysara M, Leys N, Raes J, Monsieurs P. IPED: a highly efficient denoising tool for Illumina MiSeq Paired-end 16S rRNA gene amplicon sequencing data. BMC Bioinformatics. 2016;17:192.

8. Edgar RC, Flyvbjerg H. Error filtering, pair assembly, and error correction for next-generation sequencing reads. Bioinformatics. 2015;31:3476–82.

9. Ashelford KE, Chuzhanova NA, Fry JC, Jones AJ, Weightman AJ. At least 1 in 20 16S rRNA sequence records currently held in public repositories is estimated to contain substantial anomalies. Am. Soc. Microbiol. 2005;Vol. 71.

10. Odelberg SJ, Weiss RB, Hata A, White R. Template-switching during DNA synthesis by Thermus aquaticus DNA polymerase I. Nucleic Acids Res. 1995;23:2049–57.

11. Judo MS, Wedel A B, Wilson C. Stimulation and suppression of PCR-mediated recombination. Nucleic Acids Res. 1998;26:1819–25.

12. Smyth RP, Schlub TE, Grimm A, Venturi V, Chopra A, Mallal S, et al. Reducing chimera formation during PCR amplification to ensure accurate genotyping. Gene. 2010;469:45–51.

13. Wang GC, Wang Y. The frequency of chimeric molecules as a consequence of PCR co-amplification of 16S rRNA genes from different bacterial species. Microbiology. 1996;142 (Pt 5:1107–14.

14. Wang GC, Wang YUE. Frequency of formation of chimeric molecules as a consequence of PCR coamplification of 16S rRNA genes from mixed bacterial genomes. 1997;63:4645–50.

15. Allhoff M, Schönhuth A, Martin M, Costa IG, Rahmann S, Marschall T. Discovering motifs that induce sequencing errors. BMC Bioinformatics. 2013;14 Suppl 5:S1.

16. Tsai IJ, Hunt M, Holroyd N, Huckvale T, Berriman M, Kikuchi T. Summarizing specific profiles in Illumina sequencing from whole-genome amplified DNA. DNA Res. 2014;21:243–54.

17. Schirmer M, Ijaz UZ, D'Amore R, Hall N, Sloan WT, Quince C. Insight into biases and sequencing errors for amplicon sequencing with the Illumina MiSeq platform. Nucleic Acids Res. 2015; 43:e37.

18. Kircher M, Stenzel U, Kelso J. Improved base calling for the Illumina Genome Analyzer using machine learning strategies. Genome Biol. 2009;10:R83.

19. Bentley DR, Balasubramanian S, Swerdlow HP, Smith GP, Milton J, Brown CG, et al. Accurate whole human genome sequencing using reversible terminator chemistry. Nature. 2008;456:53–9.

20. Rougemont J, Amzallag A, Iseli C, Farinelli L, Xenarios I, Naef F. Probabilistic base calling of Solexa sequencing data. BMC Bioinformatics. 2008;9:431.

21. Meyer F, Paarmann D, D'Souza M, Olson R, Glass EM, Kubal M, et al. The metagenomics RAST server - a public resource for the automatic phylogenetic and functional analysis of metagenomes. BMC Bioinformatics. 2008;9:386.

22. Schloss PD, Westcott SL, Ryabin T, Hall JR, Hartmann M, Hollister EB, et al. Introducing mothur: open-source, platform-independent, community-supported software for describing and comparing microbial communities. Appl. Environ. Microbiol. 2009;75:7537–41.

23. Caporaso JG, Kuczynski J, Stombaugh J, Bittinger K, Bushman FD, Costello EK, et al. QIIME allows analysis of high-throughput community sequencing data. Nat. Methods. 2010;7:335–6.

24. Edgar RC. Search and clustering orders of magnitude faster than BLAST. Bioinformatics. 2010;26:2460–1.

25. Hildebrand F, Tadeo R, Voigt A, Bork P, Raes J. LotuS: an efficient and user-friendly OTU processing pipeline. Microbiome. BioMed Central Ltd; 2014;2:30.

26. Fosso B, Santamaria M, Marzano M, Alonso-Alemany D, Valiente G, Donvito G, et al. BioMaS: a modular pipeline for Bioinformatic analysis of Metagenomic AmpliconS. BMC Bioinformatics. 2015;16:203.

27. Schloss PD, Gevers D, Westcott SL. Reducing the effects of PCR amplification and sequencing artifacts on 16S rRNA-based studies. PLoS One. 2011;6:e27310.

28. Mysara M, Leys N, Raes J, Monsieurs P. NoDe: a fast error-correction algorithm for pyrosequencing amplicon reads. BMC Bioinformatics. 2015;16:88.

29. Mysara M, Saeys Y, Leys N, Raes J, Monsieurs P. CATCh, an ensemble classifier for chimera detection in 16S rRNA sequencing studies. Appl. Environ. Microbiol. 2015;81:1573–84.

30. Quince C, Lanzen A, Davenport RJ, Turnbaugh PJ. Removing noise from pyrosequenced amplicons. BMC Bioinformatics. 2011;12:38.

31. Westcott SL, Schloss PD. De novo clustering methods outperform reference-based methods for assigning 16S rRNA gene sequences to operational taxonomic units. PeerJ. 2015;3:e1487.

32. Schloss PD, Westcott SL. Assessing and improving methods used in operational taxonomic unit-based approaches for 16S rRNA gene sequence analysis. Appl. Environ. Microbiol. 2011;77:3219–26.

33. Edgar RC. UPARSE: highly accurate OTU sequences from microbial amplicon reads. Nat. Methods. 2013;10:996–8.

34. Sun Y, Cai Y, Huse SM, Knight R, Farmerie WG, Wang X, et al. A large-scale benchmark study of existing algorithms for taxonomy-independent microbial community analysis. Brief. Bioinform. 2012;13:107–21.

35. Plummer E, Twin J, Bulach DM, Garland SM, Tabrizi SN. A comparison of three bioinformatics pipelines for the analysis of preterm gut microbiota using 16S rRNA gene sequencing data. J. Proteomics Bioinform. 2015;8.

36. D'Argenio V, Casaburi G, Precone V, Salvatore F. Comparative metagenomic analysis of human gut microbiome composition using two different bioinformatic pipelines. Biomed Res. Int. 2014;2014:325340.

37. Nelson MC, Morrison HG, Benjamino J, Grim SL, Graf J. Analysis, optimization and verification of Illumina-generated 16S rRNA gene amplicon surveys. PLoS One. 2014;9:e94249.

38. Pruesse E, Quast C, Knittel K, Fuchs BM, Ludwig W, Peplies J, et al. SILVA: a comprehensive online resource for quality checked and aligned ribosomal RNA sequence data compatible with ARB. Nucleic Acids Res. 2007;35:7188–96.

39. Schloss PD. The effects of alignment quality, distance calculation method, sequence filtering, and region on the analysis of 16S rRNA gene-based studies. PLoS Comput. Biol. 2010;6:e1000844.

40. Schloss PD. A high-throughput DNA sequence aligner for microbial ecology studies. PLoS One. 2009;4:e8230.

41. Schloss PD. Secondary structure improves OTU assignments of 16S rRNA gene sequences. ISME J. International Society for Microbial Ecology; 2013;7:457–60.

42. Huse SM, Welch DM, Morrison HG, Sogin ML. Ironing out the wrinkles in the rare biosphere through improved OTU clustering. Environ. Microbiol. 2010;12:1889–98.

43. Edgar RC, Haas BJ, Clemente JC, Quince C, Knight R. UCHIME improves sensitivity and speed of chimera detection. Bioinformatics. 2011;27:2194–200.

44. Bokulich NA, Subramanian S, Faith JJ, Gevers D, Gordon JI, Knight R, et al. Quality-filtering vastly improves diversity estimates from Illumina amplicon sequencing. Nat. Methods. 2013;10:57–9.

45. Magoč T, Salzberg SL. FLASH: fast length adjustment of short reads to improve genome assemblies. Bioinformatics. 2011;27:2957–63.

46. Medvedev P, Scott E, Kakaradov B, Pevzner P. Error correction of high-throughput sequencing datasets with non-uniform coverage. Bioinformatics. 2011;27:i137–41.

47. Bankevich A, Nurk S, Antipov D, Gurevich AA, Dvorkin M, Kulikov AS, et al. SPAdes: a new genome assembly algorithm and its applications to single-cell sequencing. J. Comput. Biol. 2012;19:455–77.

48. Reeder J, Knight R. Rapidly denoising pyrosequencing amplicon reads by exploiting rank-abundance distributions. Nat. Methods. 2010;7:668–9.

49. Kunin V, Engelbrektson A, Ochman H, Hugenholtz P. Wrinkles in the rare biosphere: pyrosequencing errors can lead to artificial inflation of diversity estimates. Environ. Microbiol. 2010;12:118–23.

50. Masella AP, Bartram AK, Truszkowski JM, Brown DG, Neufeld JD. PANDAseq: paired-end assembler for Illumina sequences. BMC Bioinformatics. 2012;13:31.

51. Liu B, Yuan J, Yiu S-M, Li Z, Xie Y, Chen Y, et al. COPE: an accurate k-mer-based pair-end reads connection tool to facilitate genome assembly. Bioinformatics. 2012;28:2870–4.

52. Zhang J, Kobert K, Flouri T, Stamatakis A. PEAR: a fast and accurate Illumina Paired-End reAd mergeR. Bioinformatics. 2014;30:614–20.

53. Ashelford KE, Chuzhanova NA, Fry JC, Jones AJ, Weightman AJ. At least 1 in 20 16S rRNA sequence records currently held in public repositories is estimated to contain substantial anomalies. Appl. Environ. Microbiol. 2005;71:7724–36.

54. Huber T, Faulkner G, Hugenholtz P. Bellerophon: a program to detect chimeric sequences in multiple sequence alignments. Bioinformatics. 2004;20:2317–9.

55. Haas BJ, Gevers D, Earl AM, Feldgarden M, Ward D V, Giannoukos G, et al. Chimeric 16S rRNA sequence formation and detection in Sanger and 454-pyrosequenced PCR amplicons. Genome Res. 2011;21:494–504.

56. Wright ES, Yilmaz LS, Noguera DR. DECIPHER, a search-based approach to chimera identification for 16S rRNA sequences. Appl. Environ. Microbiol. 2012;78:717–25.

57. Schloss PD, Handelsman J. Introducing DOTUR, a computer program for defining operational taxonomic units and estimating species richness. Appl. Environ. Microbiol. 2005;71:1501–6.

58. Sun Y, Cai Y, Liu L, Yu F, Farrell ML, McKendree W, et al. ESPRIT: estimating species richness using large collections of 16S rRNA pyrosequences. Nucleic Acids Res. 2009;37:e76.

59. Cai Y, Sun Y. ESPRIT-Tree: hierarchical clustering analysis of millions of 16S rRNA pyrosequences in quasilinear computational time. Nucleic Acids Res. 2011;39:e95.

60. Fu L, Niu B, Zhu Z, Wu S, Li W. CD-HIT: accelerated for clustering the next-generation sequencing data. Bioinformatics. 2012;28:3150–2.

61. Russell DJ, Way SF, Benson AK, Sayood K. A grammar-based distance metric enables fast and accurate clustering of large sets of 16S sequences. BMC Bioinformatics. 2010;11:601.

62. Ghodsi M, Liu B, Pop M. DNACLUST: accurate and efficient clustering of phylogenetic marker genes. BMC Bioinformatics. 2011;12:271.

63. Hao X, Jiang R, Chen T. Clustering 16S rRNA for OTU prediction: a method of unsupervised Bayesian clustering. Bioinformatics. 2011;27:611–8.

64. Mahé F, Rognes T, Quince C, de Vargas C, Dunthorn M. Swarm: robust and fast clustering method for amplicon-based studies. PeerJ. 2014;2:e593.

List of Tables

Table 1. Overview of the algorithms available for different steps within amplicon sequencing data analysis.

Step	Tools	Reference
paired-end assembler	FLASH	[45]
	PANDAseq	[50]
	COPE	[51]
	PEAR	[52].
Quality filtering	trim.seqs(mothur)	[6]
	split_libraries (QIIME)	[44]
	fastq_filter (USEARCH)	[8]
Denoising	Pre-cluster	[6]
	UNOISE	[8]
	IPED	[7]
Chimera Detection	Pintail	[53]
	Bellerophon	[54]
	ChimeraSlayer	[55]
	DECIPHER	[56]
	Perseus	[30]
	UPARSE	[33]
	UCHIME	[43]
	CATCh	[29]
Clustering	Dotur	[57]
	ESPRIT	[58]
	ESPRIT-Tree	[59]
	CD-HIT	[60]
	Uclust	[24]
	GramCluster	[61]
	DNAClust	[62]
	CROP	[63]
	Swarm	[64]
	UPARSE.	[33]

Table 2. The error rates for the different samples after applying various pipelines, either with complete removal of chimeric reads (via the seq.error command), or after applying the chimera removal algorithm embedded within the workflow in question.

Sample ID	Chimera Absent				Chimera removal algorithms			
	QIIME	Mothur	USEARCH	OCToPUS	QIIME	Mothur	USEARCH	OCToPUS
130403(**V34**)	0.0022	0.0006	0.0003	0.0003	0.0023	0.0008	0.0005	0.0004
130417(**V34**)	0.0018	0.0005	0.0003	0.0003	0.0019	0.0007	0.0005	0.0004
130422(**V34**)	0.0023	0.0012	0.0008	0.0009	0.0023	0.0011	0.0010	0.0009
130403(**V4**)	0.00055	0.00013	0.00010	0.00005	0.00208	0.00167	0.00161	0.00126
130417(**V4**)	0.00049	0.00010	0.00008	0.00003	0.00187	0.00150	0.00147	0.00114
130422(**V4**)	0.00048	0.00010	0.00008	0.00003	0.00182	0.00144	0.00141	0.00109
V4.I.1	0.00079	0.00007	0.00002	0.00002	0.00087	0.00013	0.00006	0.00003
V4.I.05	0.00087	0.00010	0.00002	0.00002	0.00099	0.00020	0.00008	0.00003
V4.V5.I.1	0.0257	0.0084	0.0075	0.0041	0.0241	0.0069	0.0049	0.0047
V4.V5.I.11	0.0218	0.0060	0.0072	0.0031	0.0218	0.0044	0.0047	0.0032
M1(**V34**)	0.0014	0.0006	0.0006	0.0005	0.0052	0.0039	0.0042	0.0038
M2(**V34**)	0.0014	0.0007	0.0006	0.0005	0.0058	0.0045	0.0047	0.0043
M3(**V34**)	0.0011	0.0006	0.0005	0.0005	0.0052	0.0041	0.0041	0.0039
**Average**	**0.0047**	**0.0015**	**0.0014**	**0.0008**	**0.0059**	**0.0024**	**0.0023**	**0.0019**

List of Figures

Figure 1. Overview of the different steps within each pipeline.

Figure 2. Average amount of reads removed within the various pipelines, due to improper assembly, quality filtering or chimera removal. Due to different order of the processing steps in LotuS, this pipeline could not be included in the figure (on average LotuS retains 23% of the reads).

Figure 3. Rarefaction curves of the different samples. In the X-axis the sequencing depth is given, in the Y-axis the amount of OTUs returned by each pipeline.

Figure 4. Composition of the OTUs produced via the various approaches, classified into different categories: original (blue), chimeric (violet),contaminant (green) and no hit (red). The size of the circles is representative for the amount of reads retained after running each pipeline (exact percentages can be found in supplementary file 2).

List of Supplementary materials

Supplementary File 1: Detailed description of the different mock samples and their composition

Supplementary File 2: Table illustrating the percentage of reads removed by each pipeline throughout the various samples.

Supplementary File 3: Number of OTUs per sample after being processed via the various pipelines.

Supplementary File 4: Table showing the number of OTUs per species within each sample, as well as the average number of OTUs per species (for all samples) to illustrate the over-splitting phenomenon among the various pipelines. Cells shown in black indicate missed species from the mock sample.

Supplementary File 5: Plot illustrating the computational time (in minutes) of MOCK1 samples for the three various pipelines (A), and the average computational time (in seconds) for the different steps within each pipeline (B).

Figure 1 Click here to download Figure Figure1.tif

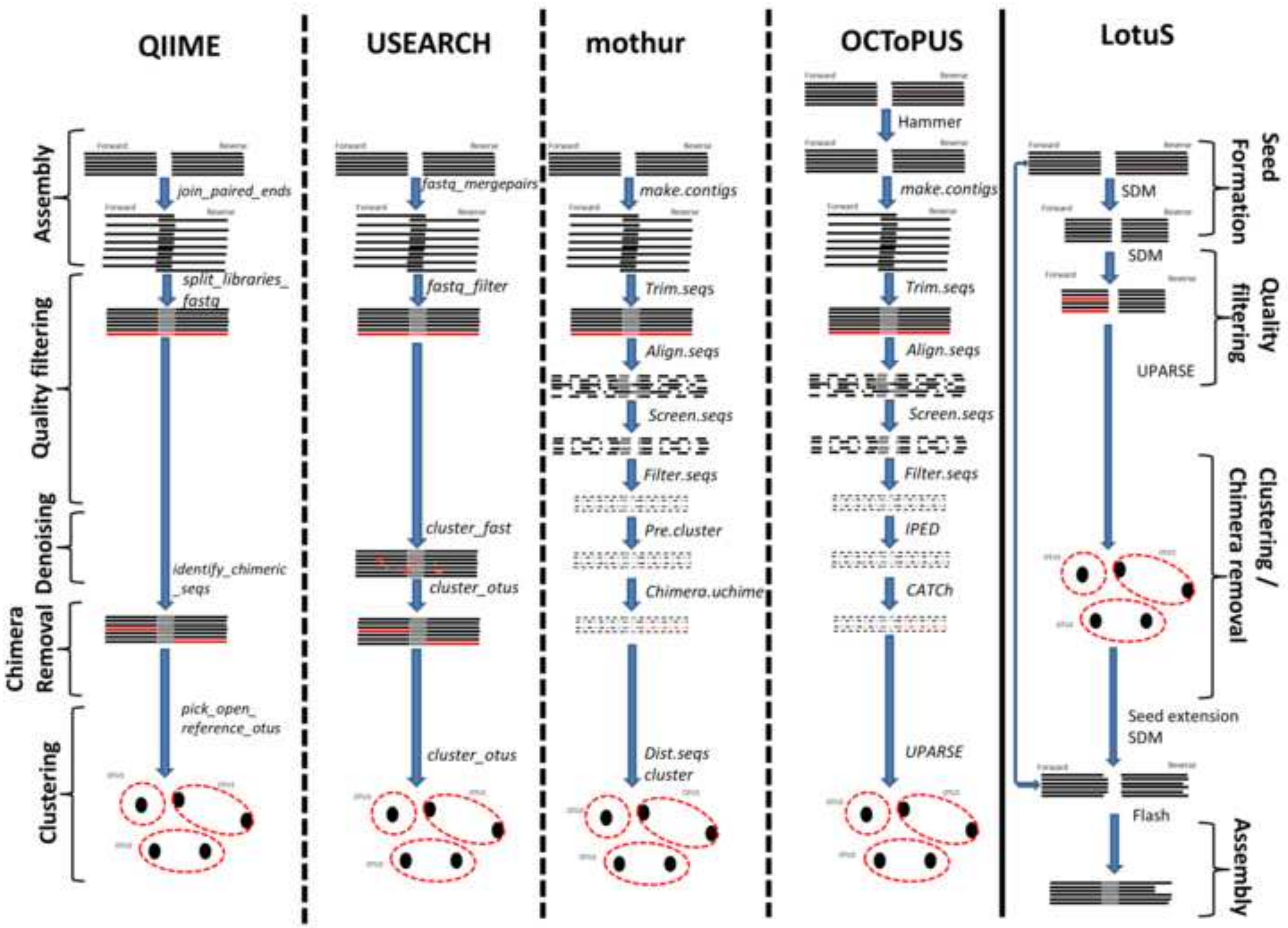

Click here to download Figure Figure2.tif

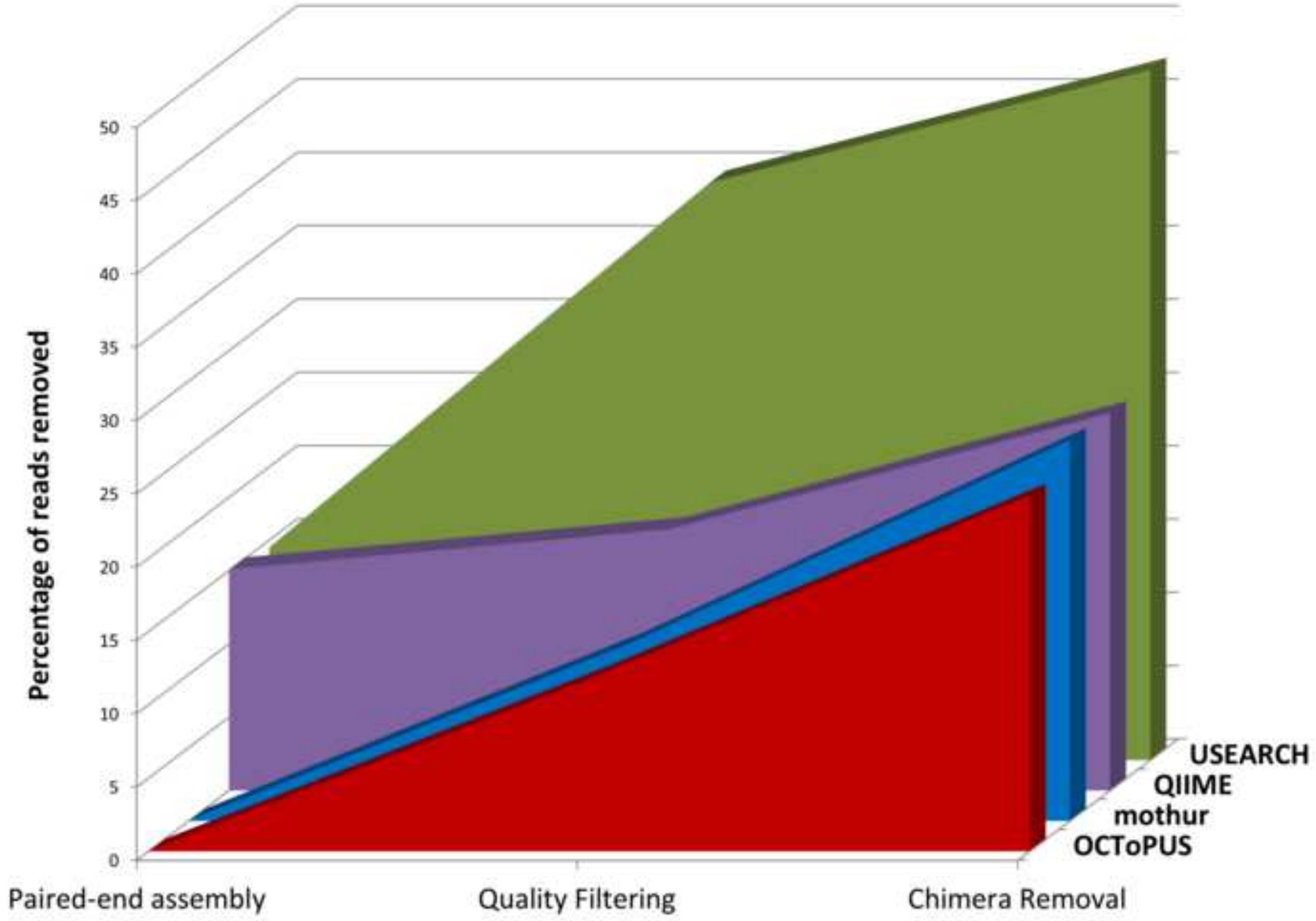

Figure 3

Click here to download Figure Figure3.tif

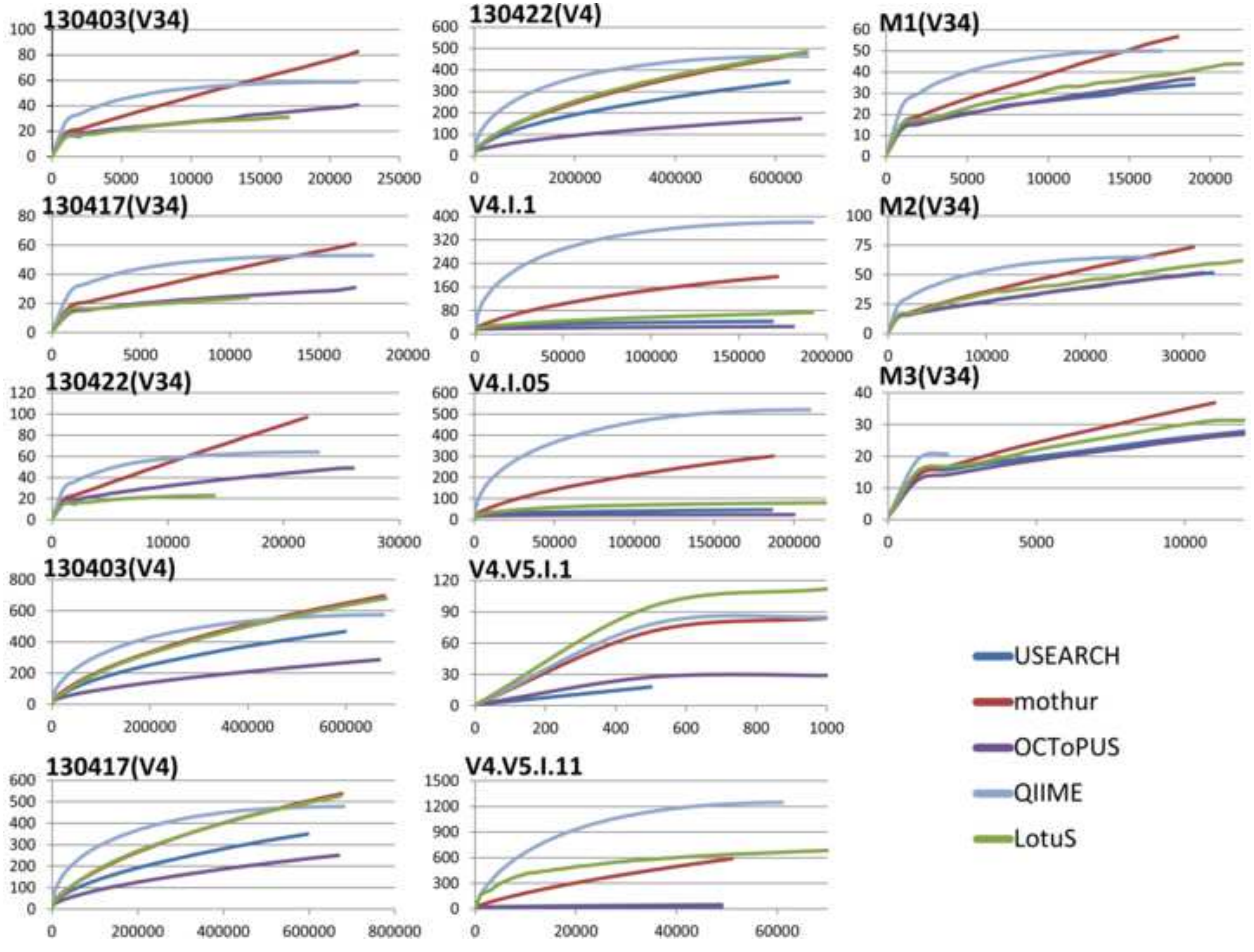

Figure 4

Click here to download Figure Figure4.tif

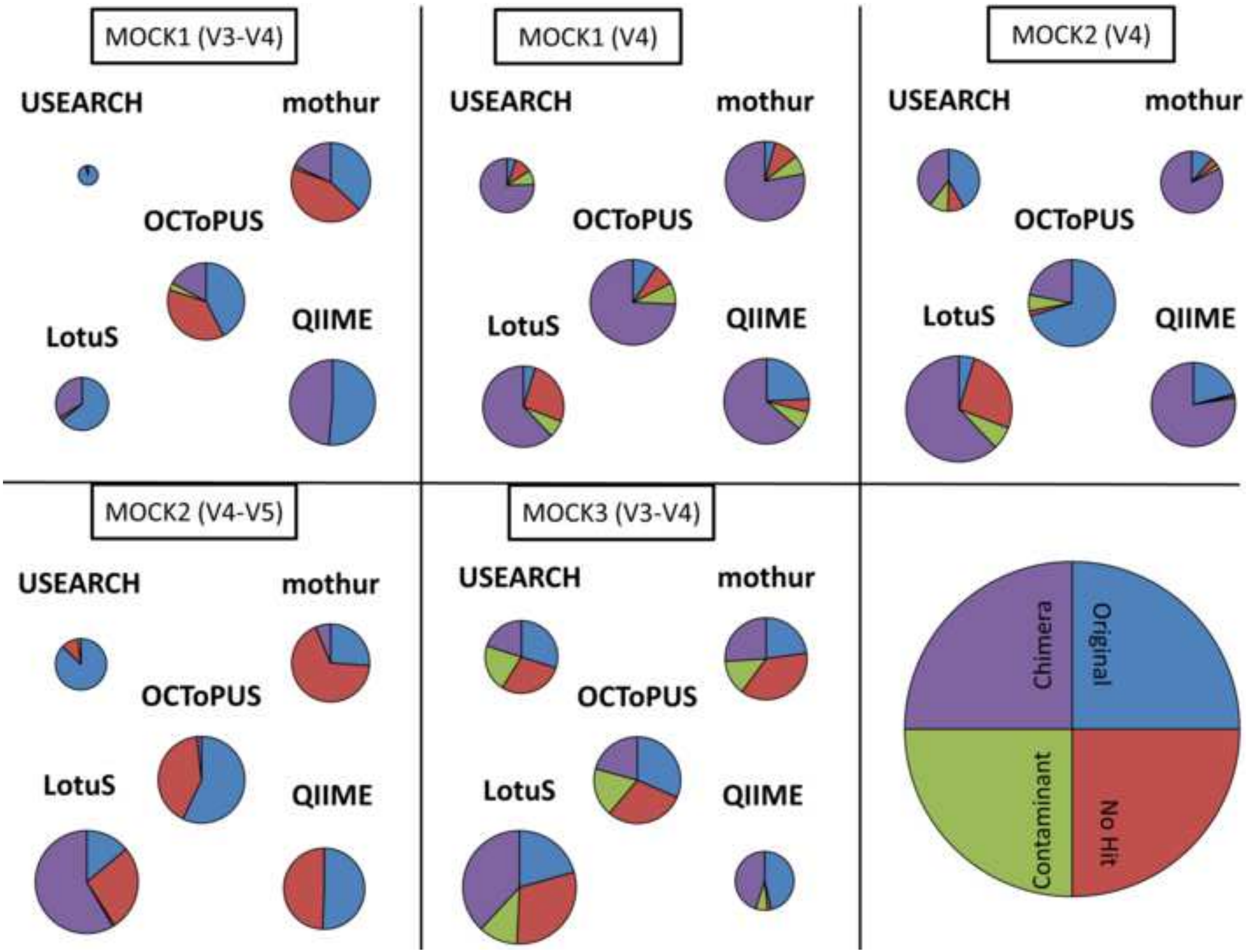

Supplementary File 1

Click here to access/download
Supplementary Material
Supplementary_File1.pdf

Supplementary File 2

Click here to access/download
Supplementary Material
Supplementary_File2.pdf

Supplementary File 3

Click here to access/download
Supplementary Material
Supplementary_File3.pdf

Supplementary File 4

Click here to access/download
Supplementary Material
Supplementary_File4.pdf

Click here to access/download
Supplementary Material
Supplementary_File5.pdf
