# Supplementary material for: From reads to operational taxonomic units: an ensemble processing pipeline for MiSeq amplicon sequencing data
Source: Gigascience. 2017 Jan 18;6(2):1–10. doi: 10.1093/gigascience/giw017 (PMC5466709; doi:10.1093/gigascience/giw017)
Supplement: Supplemental material — Additional Supplementary File 1: Detailed description of the different mock samples and their composition. Additional Supplementary File 2: Table illustrating the percentage of reads removed by each pipeline throughout the various samples. Additional Supplementary File 3: Number of OTUs per sample after being processed via the various pipelines. Additional Supplementary File 4: Table showing the number of OTUs per species within each sample, as well as the average number of OTUs per species (for all samples) to illustrate the over-splitting phenomenon among the various pipelines. Cells shown in black indicate missed species from the mock sample. Additional Supplementary File 5: Plot illustrating the computational time (in minutes) of MOCK1 samples for the three various pipelines (A), and the average computational time (in seconds) for the different steps within each pipeline (B). [file giw017_Supp.zip › Supplementary_File1.pdf]

	microbial composition	# of species	# of run	Chemistry version	16S rRNA region	Length after assembly	Length of overlapping region	Sample IDs	Source	Reference
MOCK1	*Acinetobacter baumannii* ATCC 17978,*Actinomyces odontolyticus* ATCC 17982, *Bacillus cereus* ATCC 10987, *Bacteroides vulgatus*ATCC 8482, *Clostridium beijerinckii* ATCC 51743, *Deinococcus radiodurans* ATCC 13939,*Enterococcus faecalis* ATCC 47077, *Escherichia coli* ATCC 70096, *Helicobacter pylori*ATCC 700392, *Lactobacillus gasseri* ATCC 33323, *Listeria monocytogenes* ATCC BAA-679, *Neisseria meningitidis* ATCC BAA-335, *Porphyromonas gingivalis* ATCC 33277,*Propionibacterium acnes* DSM 16379, *Pseudomonas aeruginosa* ATCC 47085, *Rhodobacter sphaeroides* ATCC 17023, *Staphylococcus aureus* ATCC BAA-1718, *Staphylococcus epidermidis* ATCC 12228, *Streptococcus agalactiae* ATCC BAA-611, *Streptococcus mutans*ATCC 700610, and *Streptococcus pneumoniae* ATCC BAA-334.	21	3	V2	V3-V4	422-428	70	130403(V34), 130417(V34), 130422(V34)	MOCK1 is available via (http://www.mothur.org/MiSeqDevelopmentData.html) under accession 130403, 130417 and 130422.	Kozich JJ, Westcott SL, Baxter NT, Highlander SK, Schloss PD. Development of a dual-index sequencing strategy and curation pipeline for analyzing amplicon sequence data on the MiSeq Illumina sequencing platform. Appl. Environ. Microbiol. 2013;79:5112–20.
					V4	251-253	250	130403(V4), 130417(V4), 130422(V4)		
MOCK2	*Acinetobacter baumannii* str. 5377, *Actinomyces odontolyticus* str. 1A.21, *Bacillus cereus* str. NRS 248, *Bacteroides vulgatus* str. NCTC 11154, *Clostridium beijerinck*ii str. NCIMB 8052, *Deinococcus radiodurans* str. R1 (smooth), *Enterococcus faecalis* str. OG1RF, *Escherichia coli str. K12 substr. MG1655, Helicobacter pylori str. 26695, Lactobacillus gasseri* str. 63 AM, *Listeria monocytogenes* str. EGDe, *Neisseria meningitidis* str. MC58, *Propionibacterium acnes* str. KPA171202, *Pseudomonas aeruginosa* str. PAO1-LAC, *Rhodobacter sphaeroides* str. ATH 2.4.1, *Staphylococcus aureus* TCH1516, *Staphylococcus epidermidis FDA* str. PCI 1200, *Streptococcus agalactiae* str. 2603 V/R, *Streptococcus mutans* str. UA159, *and Streptococcus pneumoniae* str. TIGR4.	20	2	V2	V4	251-253	250	V4.I.1, V4.I.05	MOCK2 is available via European Bioinformatics Institute Nucleotide Archive SRA under project ID PRJEB4688	Nelson MC, Morrison HG, Benjamino J, Grim SL, Graf J. Analysis, optimization and verification of Illumina-generated 16S rRNA gene amplicon surveys. PLoS One. 2014;9:e94249.
					V4-V5	390	110	V4.V5.I.1, V4.V5.I.11		
MOCK3	*Lactobacillus casei* LMG 6904, *Cupriavidus pinatubonensis* LMG 1197, *Cupriavidus metallidurans* LMG 1195, *Pseudomonas putida* LMG 24210, *Lysinibacillus spaericus* LMG 22257, *Alcanivorax borkumensis* SK2, *Alcanivorax dieselolei* 293, *Roseburia hominis* A2-138, *Geobacter sulfurreducens* DSM 12127, *Acetobacterium woodii* DSM 1030, *Porphyromonas gingivalis* ATCC 33277, *Fusobacterium nucleatum* ATCC 10953	12	1	V3	V1-V3	422-428	141	M1(V34), M3(V34) M3(V34)	National Center for Biotechnology Information SRA under project ID: SRP066114	Mysara M, Leys N, Raes J, Monsieurs P. IPED: a highly efficient denoising tool for Illumina MiSeq Paired-end 16S rRNA gene amplicon sequencing data. BMC Bioinformatics. 2016;17:192.
