# Supplementary material for: From reads to operational taxonomic units: an ensemble processing pipeline for MiSeq amplicon sequencing data
Source: Gigascience. 2017 Jan 18;6(2):1–10. doi: 10.1093/gigascience/giw017 (PMC5466709; doi:10.1093/gigascience/giw017)
Supplement: Supplemental material — Additional Supplementary File 1: Detailed description of the different mock samples and their composition. Additional Supplementary File 2: Table illustrating the percentage of reads removed by each pipeline throughout the various samples. Additional Supplementary File 3: Number of OTUs per sample after being processed via the various pipelines. Additional Supplementary File 4: Table showing the number of OTUs per species within each sample, as well as the average number of OTUs per species (for all samples) to illustrate the over-splitting phenomenon among the various pipelines. Cells shown in black indicate missed species from the mock sample. Additional Supplementary File 5: Plot illustrating the computational time (in minutes) of MOCK1 samples for the three various pipelines (A), and the average computational time (in seconds) for the different steps within each pipeline (B). [file giw017_Supp.zip › Supplementary_File2.pdf]

Percentage of rejected reads						
Sample ID		QIIME	mothur	USEARCH	LotuS	OCToPUS
**MOCK1**	130403(**V34)**	19	16	94	50	17
	130417(**V34)**	18	17	95	52	17
	130422(**V34)**	15	17	95	57	5
	130403(**V4)**	2	4	15	5	5
	130417(**V4)**	3	3	15	6	5
	130422(**V4)**	3	3	8	4	5
**MOCK2**	V4.I.1	10	19	20	6	15
	V4.I.05	13	22	22	7	17
	V4.V5.I.1	27	48	73	35	47
	V4.V5.I.11	29	43	46	6	45
**MOCK3**	M1(**V34)**	54	47	43	21	45
	M2(**V34)**	56	48	44	21	47
	M3(**V34)**	87	49	43	21	46
**Average**		**26**	**26**	**47**	**23**	**24**
