# Supplementary material for: From reads to operational taxonomic units: an ensemble processing pipeline for MiSeq amplicon sequencing data
Source: Gigascience. 2017 Jan 18;6(2):1–10. doi: 10.1093/gigascience/giw017 (PMC5466709; doi:10.1093/gigascience/giw017)
Supplement: Supplemental material — Additional Supplementary File 1: Detailed description of the different mock samples and their composition. Additional Supplementary File 2: Table illustrating the percentage of reads removed by each pipeline throughout the various samples. Additional Supplementary File 3: Number of OTUs per sample after being processed via the various pipelines. Additional Supplementary File 4: Table showing the number of OTUs per species within each sample, as well as the average number of OTUs per species (for all samples) to illustrate the over-splitting phenomenon among the various pipelines. Cells shown in black indicate missed species from the mock sample. Additional Supplementary File 5: Plot illustrating the computational time (in minutes) of MOCK1 samples for the three various pipelines (A), and the average computational time (in seconds) for the different steps within each pipeline (B). [file giw017_Supp.zip › Supplementary_File3.pdf]

	Number of OTUs												
	MOCK1						MOCK2				MOCK3		
	130403**(V34)**	130417**(V34)**	130422**(V34)**	130403**(V4)**	130417**(V4)**	130422**(V4)**	V4.I.1	V4.I.05	V4.V5.I.1	V4.V5.I.11	M1**(V34)**	M2**(V34)**	M3**(V34)**
USEARCH	16	16	15	469	351	347	44	47	18	25	34	52	28
mothur	83	61	97	697	538	478	196	302	84	590	58	74	37
OCToPUS	41	31	49	287	251	174	26	24	29	48	36	52	27
QIIME	59	53	64	575	478	465	380	521	85	1248	50	65	21
LotuS	31	24	23	679	526	487	76	81	112	724	57	79	40
