# Supplementary material for: From reads to operational taxonomic units: an ensemble processing pipeline for MiSeq amplicon sequencing data
Source: Gigascience. 2017 Jan 18;6(2):1–10. doi: 10.1093/gigascience/giw017 (PMC5466709; doi:10.1093/gigascience/giw017)
Supplement: Supplemental material — Additional Supplementary File 1: Detailed description of the different mock samples and their composition. Additional Supplementary File 2: Table illustrating the percentage of reads removed by each pipeline throughout the various samples. Additional Supplementary File 3: Number of OTUs per sample after being processed via the various pipelines. Additional Supplementary File 4: Table showing the number of OTUs per species within each sample, as well as the average number of OTUs per species (for all samples) to illustrate the over-splitting phenomenon among the various pipelines. Cells shown in black indicate missed species from the mock sample. Additional Supplementary File 5: Plot illustrating the computational time (in minutes) of MOCK1 samples for the three various pipelines (A), and the average computational time (in seconds) for the different steps within each pipeline (B). [file giw017_Supp.zip › Supplementary_File4.pdf]

MOCK1 and MOCK2		epidermidis	cereus	radiodurans	meningitidis	baumannii	beijerinckii	pylori	aeruginosa	monocytogenes	coli	mutans	faecalis	vulgatus	odontolyticus	sphaeroides	agalactiae	gasseri	gingivalis	pneumoniae	acnes	aureus
130403(V34)	USEARCH	0	1		1	1			1	1	1	1	1	1	1		1	1	1	1		1
	mothur	4	2		1	1			15	1	1	1	1	1	1		1	5	1	1		1
	OCToPUS	0	1		1	1			1	1	1	1	1	2	1		1	1	1	1		1
	QIIME	3	2		1	1			3	1	2	1	1	6	1		2	1	1	1		2
	LotuS	1	2		2	0			1	1	1	1	1	1	1		1	1	0	1		1
130417(V34)	USEARCH	0	1		1	1			1	1	1	1	1	1	1		1	1	1	1		1
	mothur	1	1		1	1			4	1	1	1	1	1	1		1	3	1	1		1
	OCToPUS	0	1		1	1			1	1	1	1	1	2	1		1	1	1	1		1
	QIIME	2	2		1	1			2	1	1	1	1	8	3		1	1	1	1		3
	LotuS	1	1		1	0			1	2	1	1	1	1	1		1	1	1	1		1
130422(V34)	USEARCH	0	1		1	1			1	1	1	1	1	1	1		1	1	1	1		1
	mothur	2	1		1	2			7	1	1	1	1	2	1		1	6	1	1		1
	OCToPUS	3	1		1	1			1	1	1	1	1	1	1		1	1	1	1		1
	QIIME	3	2		1	1			2	1	2	1	1	7	2		1	1	1	2		3
	LotuS	2	2		1	0			1	1	1	1	1	1	1		1	1	1	1		1
130403(V4)	USEARCH	1	1	1	1	1	1	1	1	1	1	1	1	1	1	1	1	1	1	1	1	
	mothur	1	0	2	1	1	1	1	1	1	1	1	1	1	1	1	1	1	1	1	1	
	OCToPUS	1	1	2	1	1	1	1	1	1	1	1	1	1	1	1	1	1	1	1	1	
	QIIME	21	7	1	1	3	2	4	8	1	14	2	13	19	2	2	5	9	1	2	2	
	LotuS	2	1	1	1	1	1	2	1	1	0	0	3	2	1	1	1	1	1	0	1	
130417(V4)	USEARCH	1	1	1	1	1	1	1	1	1	1	1	1	1	1	1	1	1	1	1	1	
	mothur	1	3	2	1	1	1	1	1	2	1	1	1	1	1	1	1	1	1	1	1	
	OCToPUS	1	1	1	1	1	1	1	1	1	1	1	1	1	1	1	1	1	1	1	1	
	QIIME	21	7	1	1	4	2	4	9	1	13	1	12	17	2	2	6	11	1	4	2	
	LotuS	4	1	1	1	2	1	1	2	5	1	2	3	3	0	1	2	1	1	0	0	
130422(V4)	USEARCH	1	1	1	1	1	1	1	1	2	1	1	1	1	1	1	1	1	1	1	1	
	mothur	2	2	2	1	1	1	1	1	2	1	1	1	2	1	1	1	1	1	1	1	
	OCToPUS	2	1	1	1	1	1	1	1	1	1	1	1	2	1	1	1	1	1	1	1	
	QIIME	21	8	1	1	4	2	4	7	1	15	2	12	17	2	3	6	11	2	3	2	
	LotuS	2	0	1	1	1	2	1	2	4	0	0	3	3	1	1	1	1	1	0	0	
V4.I.1	USEARCH	1	1	1	1	1	1	1	1	1	1	1	1	1	1	1	1	1		1	1	
	mothur	4	4	2	1	1	1	1	1	1	1	2	2	1	1	1	1	1		1	1	
	OCToPUS	1	1	1	1	1	1	1	1	1	1	1	0	1	1	1	1	1		0	1	
	**QIIME**	**22**	**4**	**1**	**1**	**2**	**2**	**2**	**1**	**2**	**7**	**1**	**10**	**9**	**2**	**1**	**6**	**7**		**6**	**1**	
	LotuS	2	1	1	1	2	1	0	1	1	1	1	2	1	1	1	1	0		1	1	
V4.I.05	USEARCH	1	1	1	1	1	1	1	1	1	1	1	1	1	1	1	1	1		1	1	
	mothur	2	3	1	1	1	1	1	1	1	1	1	3	2	1	1	1	1		1	1	
	OCToPUS	1	1	1	1	1	1	1	1	1	1	1	0	1	1	1	1	1		1	1	
	**QIIME**	**23**	**3**	**1**	**1**	**3**	**1**	**4**	**2**	**2**	**6**	**2**	**14**	**10**	**2**	**1**	**5**	**8**		**9**	**2**	
	LotuS	3	2	1	1	1	3	0	1	1	1	1	1	1	1	1	0	0		1	1	
V4.V5.I.1	USEARCH	1	1	1	1	1	1	1	0	2	1	1	1	1	1	1	1	0		0	1	
	mothur	2	4	1	2	1	1	2	1	3	1	1	2	1	1	1	3	2		3	1	
	OCToPUS	1	1	0	1	1	1	1	1	2	1	1	1	1	1	1	1	2		2	1	
	**QIIME**	**12**	**7**	**1**	**2**	**2**	**2**	**2**	**1**	**4**	**2**	**5**	**6**	**5**	**2**	**1**	**3**	**2**		**2**	**3**	
	LotuS	1	1	0	0	1	2	0	0	2	0	2	2	2	1	3	0	0		1	1	
V4.V5.I.11	USEARCH	1	2	1	1	1	1	1	1	1	1	1	1	1	1	1	1	1		1	1	
	mothur	12	15	1	4	3	3	3	1	7	1	2	8	3	1	1	2	4		3	2	
	OCToPUS	1	2	1	1	1	1	1	1	1	1	1	1	1	1	1	1	1		1	1	
	**QIIME**	**57**	**38**	**3**	**7**	**5**	**12**	**3**	**4**	**39**	**10**	**18**	**33**	**12**	**8**	**10**	**28**	**15**		**12**	**10**	
	LotuS	9	6	7	2	2	9	5	1	3	1	8	4	5	3	3	3	2		1	5	

Average number of OTUs per species over all samples (over split OTUs) MOCK1 and MOCK2																						
**Average**		epidermidis	cereus	radiodurans	meningitidis	baumannii	beijerinckii	pylori	aeruginosa	monocytogenes	coli	mutans	faecalis	vulgatus	odontolyticus	sphaeroides	agalactiae	gasseri	gingivalis	pneumoniae	acnes	aureus
1.0	USEARCH	1	1	1	1	1	1	1	1	1	1	1	1	1	1	1	1	1	1	1	1	1
1.7	mothur	3	4	2	1	1	1	1	3	2	1	1	2	2	1	1	1	3	1	1	1	1
1.0	OCToPUS	1	1	1	1	1	1	1	1	1	1	1	1	1	1	1	1	1	1	1	1	1
5.2	QIIME	19	8	1	2	3	3	3	4	5	7	3	10	11	3	3	6	7	1	4	3	3
1.4	LotuS	3	2	2	1	1	3	1	1	2	1	2	2	2	1	2	1	1	1	1	1	1

MOCK3		Mock_Mock_16s_M17	Mock_Mock_16s_M18	Mock_Mock_16s_M1	Mock_Mock_16s_M16	Mock_Mock_16s_M7	Mock_Mock_16s_M11	Mock_Mock_16s_M6	Mock_Mock_16s_M2	Mock_Mock_16s_M15	Mock_Mock_16s_M9	Mock_Mock_16s_M10	Mock_Mock_16s_M3
M1(V34)	USEARCH	1	1	1	1	1	1	0	1	1	1	1	
	mothur	1	1	1	2	1	1	1	1	1	1	2	
	OCToPUS	1	1	1	2	1	1	0	1	1	1	2	
	QIIME	1	4	2	2	1	3	1	2	1	1	2	
	LotuS	1	0	1	1	0	2	1	1	1	1	2	
M2(V34)	USEARCH	1	1	1	1	1	1	1	1	1	1	1	
	mothur	1	1	1	1	1	2	1	1	2	1	2	
	OCToPUS	1	1	1	1	1	1	1	1	1	1	1	
	QIIME	3	3	2	2	1	8	1	1	1	1	1	
	LotuS	1	1	2	2	1	1	1	2	1	0	2	
M3(V34)	USEARCH	1	1	1	1	1	1	1	1	1	1	1	
	mothur	1	1	0	1	1	1	0	1	1	1	1	
	OCToPUS	1	1	1	1	1	1	1	1	1	1	1	
	QIIME	1	2	1	1	1	2	1	1	1	1	1	
	LotuS	1	1	1	1	1	1	0	1	1	1	1	
Average number of OTUs per species over all samples (over split OTUs)													
**Average**		Mock_Mock_16s_M17	Mock_Mock_16s_M18	Mock_Mock_16s_M1	Mock_Mock_16s_M16	Mock_Mock_16s_M7	Mock_Mock_16s_M11	Mock_Mock_16s_M6	Mock_Mock_16s_M2	Mock_Mock_16s_M15	Mock_Mock_16s_M9	Mock_Mock_16s_M10	Mock_Mock_16s_M3
1.0	USEARCH	1	1	1	1	1	1	0	1	1	1	1	0
1.1	mothur	1	1	1	1	1	1	1	1	1	1	2	0
1.0	OCToPUS	1	1	1	1	1	1	0	1	1	1	1	0
1.7	QIIME	2	3	2	2	1	4	1	1	1	1	1	0
1.1	LotuS	1	1	1	1	1	1	1	1	1	1	2	0
