# Supplementary material for: From reads to operational taxonomic units: an ensemble processing pipeline for MiSeq amplicon sequencing data
Source: Gigascience. 2017 Jan 18;6(2):1–10. doi: 10.1093/gigascience/giw017 (PMC5466709; doi:10.1093/gigascience/giw017)
Supplement: Supplementary file 11 [file giw017_Response_to_Reviewer_Commnets_Revison_2.pdf]

Added DOI details.
