# Supplementary material for: From reads to operational taxonomic units: an ensemble processing pipeline for MiSeq amplicon sequencing data
Source: Gigascience. 2017 Jan 18;6(2):1–10. doi: 10.1093/gigascience/giw017 (PMC5466709; doi:10.1093/gigascience/giw017)
Supplement: Response_to_Reviewer_Comments_Revision_1.pdf [file giw017_Response_to_Reviewer_Comments_Revision_1.pdf]

Reviewer #1: Thank you very much for your detailed answers to my previous remarks, they are quite convincing. While I think the manuscript can be accepted for publication in its current form, I still think it would benefit from a better choice of evaluation criteria. While I understand the adoption of error rate for sequence data, I think the richness (number of OTUs) is not informative enough and it should be complemented with ecological measures such as, for instance, the diversity (Shannon-Wiener index) used in http://bib.oxfordjournals.org/content/12/6/614 to compare OTU clustering results from MOTHUR and QIIME.

We followed the suggestion of the reviewer, and calculated the theoretical diversity index of the mock community, as well as for the different tested pipelines, and this for a wide range of diversity indices. However, in general, the diversity indices obtained for the different pipelines are all floating around the same value, with minor difference compared with the theoretical values. When zooming in on the individual values, QIIME is in most cases the closest to the theoretical values. This is somewhat contradictory, as QIIME has certainly not the lowest sequencing error rate, and also contains a high number of spurious OTUs (e.g., a significant amount of chimeric sequences, which is higher than the number obtained for USEARCH or OCToPUS).

Where we agree with the reviewer that this type of analysis is meaningful in case of a real-life community (or a simulated dataset with high diversity as constructed in the article recommended by the reviewer), it seems to be less useful in case of mock communities. This might also explain why this evaluation criterion (based on diversity indices) is not frequently used by researchers testing algorithms or pipelines on mock communities in the context of 16S rRNA amplicon sequencing studies.

Table 1: Overview of the Shannon Index (upper table) and Inverse Simpson index (lower table) for each mock community and each analysis pipeline, as well as the theoretical value.

Shannon	OCToPUS	USEARCH	LotuS	QIIME	mothur	Reference
MCK1_V34_03	2,330	2,259	2,257	2,649	2,350	2,996
MCK1_V34_17	2,314	2,231	2,210	2,629	2,308	2,996
MCK1_V34_22	2,491	2,358	2,237	2,735	2,437	2,996
MCK1_V4_03	2,786	2,791	2,792	2,956	2,829	2,996
MCK1_V4_17	2,785	2,784	2,793	2,951	2,824	2,996
MCK1_V4_22	2,808	2,816	2,818	2,959	2,835	2,996
MCK2_V4_1	2,505	2,675	2,692	3,022	2,693	2,944
MCK2_V4_105	2,606	2,696	2,714	3,086	2,720	2,944
MCK2_V45_1	2,154	2,503	3,612	3,293	2,888	2,944
MCK2_V45_11	2,643	2,612	4,152	3,972	2,826	2,944
M1	2,284	2,370	2,415	2,558	2,372	2,485
M2	2,376	2,389	2,436	2,559	2,379	2,485
M3	2,316	2,323	2,429	2,436	2,325	2,485

Inv. simpson	OCToPUS	USEARCH	LotuS	QIIME	mothur	Reference
MCK1_V34_03	0,116	0,140	0,131	0,089	0,120	0,050
MCK1_V34_17	0,115	0,143	0,139	0,090	0,125	0,050
MCK1_V34_22	0,096	0,118	0,132	0,082	0,105	0,050
MCK1_V4_03	0,071	0,073	0,073	0,067	0,070	0,050
MCK1_V4_17	0,071	0,073	0,073	0,067	0,070	0,050
MCK1_V4_22	0,069	0,070	0,070	0,066	0,069	0,050
MCK2_V4_1	0,092	0,081	0,079	0,070	0,082	0,052
MCK2_V4_105	0,084	0,079	0,077	0,067	0,080	0,052
MCK2_V45_1	0,176	0,095	0,043	0,065	0,080	0,052
MCK2_V45_11	0,086	0,089	0,033	0,046	0,083	0,052
M1	0,117	0,108	0,100	0,096	0,110	0,083

M2	0,107	0,105	0,097	0,095	0,108	0,083
M3	0,109	0,108	0,096	0,106	0,109	0,083
