# Supplementary material for: From reads to operational taxonomic units: an ensemble processing pipeline for MiSeq amplicon sequencing data
Source: Gigascience. 2017 Jan 18;6(2):1–10. doi: 10.1093/gigascience/giw017 (PMC5466709; doi:10.1093/gigascience/giw017)
Supplement: Response_to_Reviewer_Comment_Original_Submission.pdf [file giw017_Response_to_Reviewer_Comment_Original_Submission.pdf]

Reviewer #1:

REMARK 1) The authors cite several state-of-the-art pipelines: MG-RAST, mothur, QIIME, USEARCH, LotuS, BioMaS. However, they limit their comparative study to mothur, QIIME, USEARCH and LotuS, without any justification. I would urge them to also include MG-RAST and BioMaS in their study. In particular, the latter has been shown to produce the most accurate results in the benchmark presented in [26], and I would expect similar results on the three mock communities adopted for this manuscript.
BioMaS and MG-RAST are indeed two intensively used state-of-the-art pipelines. However, we actually omitted both approaches in our comparison due to different reasons.

For BioMaS, the main issue is the fundamental difference with the other pipelines from a conceptual point of view, as it is following a phylotyping-based approach. This implies that sequences are grouped based on their sequence-similarities to a reference taxonomy database, rather than based on their sequence-similarity to each other (i.e. OTU clustering), as such jeopardizing the fairness of the comparison as we are assessing OTUs rather than phylotypes. Indeed, the latter OTU clustering approach is integrated in the most widely used bioinformatics pipelines like mothur [1], QIIME [2], MG-RAST [3] and UPARSE [4]. Such a skewed comparison would make our evaluation criteria obsolete, such as the number and composition of the OTUs. Moreover, such a binning procedure will be biased towards the existing taxonomic classification – inheriting its classification errors – which is largely based on cultivable organisms [5–7]. Although QIIME and mothur do include a phylotyping approach in their software, they do not recommend using it (e.g., http://www.mothur.org/wiki/454_SOP). BioMaS has been shown to produce the most accurate results compared to mothur and QIIME, however we assume that the authors of BioMaS [8] used the less recommended phylotyping approach for mothur and QIIME to make it a fair comparison (the authors of BioMaS did not specify the exact commands used for these pipelines in their manuscript).

Concerning MG-RAST, this tool does not include a chimera detection step, which would lead to a dramatic inflation in the number of OTUs. The absence of such an important aspect makes it difficult to integrate this pipeline in the comparison. Integrating an external chimera detection approach in the MG-RAST pipeline would not allow us to claim our approach as "black-box" testing. In the comparison done between QIIME and MG-RAST by D'Argenio et al. (2014), taxonomic assignment was more accurate when using QIIME, which in turn improved the diversity analysis output. Additionally, a dramatic inflation of computational time was observed for the server-based MG-RAST compared to QIIME (~ 50-fold), which was also reported in the comparison published in Plummer et al [10].

Nonetheless, as a proof of principle we have conducted a small prototype experiment using the MOCK1 V34 samples. BioMaS does not report in its output the exact number of phylotypes at species level, and only outputs the first 60 phylotypes while grouping together all less abundant phylotypes. We tried to include MG-RAST into the comparative analysis yet they experience some technical problems with their server so that we only managed to upload the data. In all samples, BioMaS produced more than 60 species, which is more than the number of OTUs calculated via LotuS, USEARCH, QIIME and OCToPUS (see below).

Table 1 Number of OTUs or Phylotypes produced after various processing pipelines.

	Number of OTUs/Phylotypes		
MOCK1	V34-130403	V34-130417	V34-130422
USEARCH	16	16	15
MOTHUR	83	61	97
OCTOPUS	41	31	49
QIIME	59	53	64
LotuS	31	24	23
BioMaS	60+	60+	60+

REMARK 2) The choice of benchmark data is not properly justified, and there is no detailed description of the three mock communities in terms of their complexity. Previous benchmarks have focused on covering low-complexity (one dominant species), mid-complexity (several dominant species) and high-complexity (no dominant species) microbial communities.

There are three options for the selection of benchmark data: a simulated data set, a real biological sample or a mock sample. Simulated data will fail to take into account the complexity of the contributing factors in real-life (PCR bias, sequencing errors, chimeras, etc.) and real biological data will not allow for an accurate comparison between the tools as the exact composition of the community is not known. Therefore, we choose for mock samples as this is to our opinion most optimal approach, and which is a choice supported by many researchers conducting comparative analysis in this field [11–14].

For the construction of mock samples, researchers used different strategies: some add their bacterial DNA with a different level of concentrations. However, due to the biases in the process (e.g., PCR bias, influence of the DNA extraction, number of paralogs within a species), there are significant discrepancies between the designed abundances and measured read abundances, see e.g. Turnbaugh et al (PMID 20363958) Fig S4 and Edgar et al. (PMID 23955772) Table SN1.2 where several species were absent or represented by only one or a few reads. This will jeopardize the integrity of the comparative analysis. Thus, as the usefulness of such complexity is questionable, we prefer to use another strategy by using even mock communities, similar to [11,15,16]. However, the mock communities were challenging in the sense that they have high species diversity, yet some of the species are taxonomically closely related and share a high sequence similarity (added to the mock community in order to assess the over-clustering phenomenon).

Indeed we did not provide the exact composition of the mock communities, as we relied on the original publication, yet we added extra information on the composition of the mock communities and a detailed description was added as an additional file 1. Moreover, we also added in the text a motivation why we have chosen for a mock sample, at line 114-118.

REMARK 3) The choice of evaluation criteria is not properly justified either. In particular, the error rate is probably not the most informative index and, in any case, is not even defined in this manuscript.

As mentioned in our justification, we wanted to address this comparative analysis as a black box, meaning that we did not intend to compare the individual algorithms included in the different pipelines, such as algorithms for clustering, denoising, chimera detection, etc. (as numerous papers are available in the field performing those benchmarks) but rather focus on the end-result of the pipelines (see Fig 1 in the manuscript). As the output of the pre-processing steps are cleaned-up reads, and the output of the processing steps is an OTU table (also known as shared file, biom file or OTU count table) with the corresponding OTU sequences, we evaluate the error rate (as the end product of the pre-processing steps) as well as the OTU count and composition (as the end step of the processing steps).

Although the number of OTUs has commonly been used by others as an evaluation parameter for the sequencing quality [11–13,17–19], application of this approach is generally flawed, because the number of spurious OTUs is affected by the amount of reads and the level of complexity within the mock samples [17]. The error rates on the other hand provide the most accurate assessment of the ability of the pre-processing steps to remove erroneous bases. Being unaffected by the complexity of the mock, it provides a clearer picture of the amount of errors to be expected in a real (non-mock) biological sample. As a result, many researchers in the field preferred the use of the error rate in the evaluation of their 16S rRNA amplicon sequencing algorithms [4,13,14,17,18,20].

A last argument to include the error rate as an evaluation criterion is the fact that the inability of analysis pipelines to adequately handle sequencing errors will result in an increase in the error rate, which will be propagated in the number of spurious OTUs, as mentioned the work of Edgar [4] "This shows that the large number of OTUs is primarily due to high read error rates, especially towards the end of the sequence, as

quality tends to drop as the position increases (Fig. SN1.1, lower plot, see also Fig. SN3.1)."

We agree with the reviewer that the definition of "error rate" was not addressed in the manuscript. Therefore we added the description in line 208-209.

Reviewer #2:

Overview: Mysara et al. compare 4 different pipelines based on reads throughput, error rate and OTU accuracy. They also introduce a novel pipeline called OCToPUS (Optimized CATCh, mother, IPED, UPARSE, and SPAdes) as an alternative to these pipelines. Their approach is to incorporate beneficial aspects of various tools into a novel pipeline which will then provide an end result which is superior to any of the previous tools / pipelines used alone.

The text of the paper is very readable and well written in terms of language and flow for the paper.
We thank the reviewer for the appreciation of our text.

REMARK 1: As the authors state this is a "fast-evolving discipline". As such, there are recently released software and pipelines that address some of the issues and challenges that the authors address in this paper.
Would like to have seen a discussion by the authors as to why they chose the particular parts from the other tools and algorithms, i.e. what were the presumed benefits gained from implementing those approaches in their new pipeline. And did the results support their reasons for choosing those particular parts from the different pipelines?

Numerous tools have been developed to tackle the individual challenges of 16S rRNA amplicon sequencing data analysis, such as denoising, quality filtering, chimera detection and OTU clustering. A few of these tools have been successfully incorporated in different pipelines. On the other hand, some of these tools have not been incorporated in these pipelines. Here, we aim at providing a one-stop solution incorporating various tools guided only by the end results. Except for the pre-assembly quality filtering steps, for all algorithms we could rely on scientific literature to justify the selected tools based on their outstanding performance (see below).
- When developing the OCToPUS pipeline, we tested the idea of incorporating a pre-assembly quality filtering step. Despite the fact that this step is not yet been established in most state-of-the-art pipelines, evaluation of the end results of our analysis pipeline showed a significant beneficial effect on the end result. Integrating HAMMER as pre-assembly quality-filtering step led to a reduction of the error rate by 5%, which consequently lead to a reduction of the number of spurious OTUs by 9%.
- For the denoising step, IPED was able to correct double the amount of errors and significantly reduce the number of spurious OTUs compared to other algorithms available [21].
- For the removal of chimeric sequences, plenty of tools have been developed, each of them showing specific benefits when dealing with challenging chimeras: e.g, UCHIME showed robustness against the presence of sequencing errors, ChimeraSlayer clearly demonstrated a clear advantage when dealing with sequences containing indels at low divergence level, and DECIPHER performed very efficient on chimera with a short chimeric range. CATCh combines all these advantages into one ensemble algorithm, thereby incorporating all individual predictions into one combined score. As such, applying CATCh, has been found to increase the sensitivity (i.e., detecting true chimera) with 8% without affecting the specificity (i.e., wrongly identifying a correct sequence as chimeric), as illustrated in [22].
- Concerning the OTU clustering step, UPARSE has been proven to outperform the other state-of-the-art algorithms, bringing the number of OTUs closer to the actual number of species [4].
- For assembling the pipeline, we used the mothur software pipeline as backbone, in which we replaced the default program by our own selection (IPED, CATCh and UPARSE), or in which we plugged an extra algorithm with beneficial effect (HAMMER).

We agree with the reviewer that we did not cover the justification of the choice of the tools within our discussion thoroughly. Therefore we extensively discussed this point in the results and discussion [line 232-248].

REMARK 2: Also, the authors state the work "rather treats the entire pipeline as a black box and assesses the accuracy using a unified evaluation process..." But it seems then that deactivation of various commands in some of the other pipelines that were being compared might influence that end result and therefore be an unfair comparison. If indeed the authors wanted to compare results at the end of each pipeline, I would have preferred seeing a comparison of the results based on the default settings and final outcome from the various pipelines.

We agree with the reviewer that it was our intention to treat each of the pipelines as “black box”, and we also adhered to this rule for almost all parameter settings. The only part where we changed the default parameters is in those steps where ad-hoc filtering steps were performed i.e. removal of singletons in the USEARCH, LotuS and OCToPUS pipeline (implementing the UPARSE clustering algorithm) and taxonomic anomalies in the mothur and LotuS pipeline. Similar changes were applied in the comparative analysis done by LotuS [23], where the removal of taxonomic abnormalities was deactivated, and in the analysis done by Edgar [4] where the results without the removal of singletons was presented, to allow a fair comparison with other alternatives.

It is important to stress that the removal of singletons and taxonomic anomalies is - one way or another- included in all pipelines, but only in the last step (post-processing), i.e. after creating the OTU table. For instance, in mothur singletons are handled in the subsampling step, while QIIME requires a minimum count of the OTUs thereby excluding the rare OTUs in the post-processing phase. However, as indicated in our title and throughout the manuscript, our comparative analysis is only dealing with the first two phases (pre-processing and processing) making the number of singletons and the presence of taxonomic anomalies relevant criteria in our benchmark study.

Failure of the pipelines to remove chimera, or inadequately handle sequencing errors, would ultimately result in more spurious OTUs (singletons mainly among others) with abnormalities in their taxonomic classification. Therefore, omitting these steps is mandatory for the fairness of our comparison. We address this issue in the manuscript at line 137-142.

Level of interest:
This is an article of interest to a field that is still working on developing appropriate and accurate analysis and pipelines for microbial sequence data.

Quality of written English
Acceptable; see minor editing comments

Minor editing comments:
We wish to thank the reviewer for pointing out these comments, we have addressed all issued mentioned below accordingly.

Table 2
Sample ID - 'v4.v5.1' should be v4.v5.I1

Line 43 - high "through-put" should be high-throughput to keep consistent with rest of text
Line 52 - need space between "IonTorrent"
Line 83 - double commas
Line 95 - sentence doesn't make sense; remove "be"
Line 96 - term should be plural

References
Line 338: Ion Torrent should be capitalized
Line 341: Illumina needs first letter capitalized
Line 352-354: Title is in title case as compared to other references; just needs consistency among references
Line 357: The "a" should be capitalized
Line 364-365: Reference in title case
Line 370: Missing page numbers for article
Line 411: Reference in title case
Line 400 and Line 442 - Quince reference is duplicated
Line 448 - Illumina should be capitalized
Line 387 and Line 471 - Edgar reference is duplicated
24. Edgar RC. Search and clustering orders of magnitude faster than BLAST. Bioinformatics. 2010;26:2460-1.
62. Edgar RC. Search and clustering orders of magnitude faster than BLAST. Bioinformatics. 2010;26:2460-1.

Line 484 - should Table 1 legend be in a different font?
Line 489 - should Table 2 legend be in a different font?

Line 505-507 - in a different font

Figure 1 - "Mothur" is capitalized
Figure 2 - "Mothur" is capitalized; OCTOPUS is all caps
Figure 3 - "Mothur" is capitalized
Figure 4 - "Mothur" is capitalized

References
1. Schloss PD, Westcott SL, Ryabin T, Hall JR, Hartmann M, Hollister EB, et al. Introducing mothur: open-source, platform-independent, community-supported software for describing and comparing microbial communities. Appl. Environ. Microbiol. 2009;75:7537–41.
2. Caporaso JG, Kuczynski J, Stombaugh J, Bittinger K, Bushman FD, Costello EK, et al. QIIME allows analysis of high-throughput community sequencing data. Nat. Methods. 2010;7:335–6.
3. Meyer F, Paarmann D, D'Souza M, Olson R, Glass EM, Kubal M, et al. The metagenomics RAST server - a public resource for the automatic phylogenetic and functional analysis of metagenomes. BMC Bioinformatics. 2008;9:386.
4. Edgar RC. UPARSE: highly accurate OTU sequences from microbial amplicon reads. Nat. Methods. 2013;10:996–8.
5. Amann RI, Ludwig W, Schleifer KH. Phylogenetic identification and in situ detection of individual microbial cells without cultivation. Microbiol. Rev. 1995;59:143–69.
6. Rosselló-Móra R. Towards a taxonomy of Bacteria and Archaea based on interactive and cumulative data repositories. Environ. Microbiol. 2012;14:318–34.
7. Wang Q, Garrity GM, Tiedje JM, Cole JR. Naive Bayesian classifier for rapid assignment of rRNA sequences into the new bacterial taxonomy. Appl. Environ. Microbiol. 2007;73:5261–7.
8. Fosso B, Santamaria M, Marzano M, Alonso-Alemany D, Valiente G, Donvito G, et al. BioMaS: a modular pipeline for Bioinformatic analysis of Metagenomic AmpliconS. BMC Bioinformatics. 2015;16:203.
9. D'Argenio V, Casaburi G, Precone V, Salvatore F. Comparative metagenomic analysis of human gut microbiome composition using two different bioinformatic pipelines. Biomed Res. Int. 2014;2014:325340.
10. Plummer E, Twin J, Bulach DM, Garland SM, Tabrizi SN. A Comparison of Three Bioinformatics Pipelines for the Analysis of Preterm Gut Microbiota using 16S rRNA Gene Sequencing Data. J. Proteomics Bioinform.

2015;8.
11. Schloss PD, Gevers D, Westcott SL. Reducing the effects of PCR amplification and sequencing artifacts on 16S rRNA-based studies. PLoS One. 2011;6:e27310.
12. Edgar RC, Flyvbjerg H. Error filtering, pair assembly, and error correction for next-generation sequencing reads. Bioinformatics. 2015;31:3476–82.
13. Quince C, Lanzen A, Davenport RJ, Turnbaugh PJ. Removing noise from pyrosequenced amplicons. BMC Bioinformatics. BioMed Central Ltd; 2011;12:38.
14. Huse SM, Welch DM, Morrison HG, Sogin ML. Ironing out the wrinkles in the rare biosphere through improved OTU clustering. Environ. Microbiol. 2010;12:1889–98.
15. Schloss PD, Jenior ML, Koumpouras CC, Westcott SL, Highlander SK. Sequencing 16S rRNA gene fragments using the PacBio SMRT DNA sequencing system. PeerJ. PeerJ Inc.; 2016;4:e1869.
16. Nelson MC, Morrison HG, Benjamino J, Grim SL, Graf J. Analysis, optimization and verification of Illumina-generated 16S rRNA gene amplicon surveys. PLoS One. 2014;9:e94249.
17. Kozich JJ, Westcott SL, Baxter NT, Highlander SK, Schloss PD. Development of a dual-index sequencing strategy and curation pipeline for analyzing amplicon sequence data on the MiSeq Illumina sequencing platform. Appl. Environ. Microbiol. 2013;79:5112–20.
18. Reeder J, Knight R. Rapidly denoising pyrosequencing amplicon reads by exploiting rank-abundance distributions. Nat. Methods. 2010;7:668–9.
19. Kunin V, Engelbrektson A, Ochman H, Hugenholtz P. Wrinkles in the rare biosphere: pyrosequencing errors can lead to artificial inflation of diversity estimates. Environ. Microbiol. 2010;12:118–23.
20. Schloss PD, Westcott SL. Assessing and improving methods used in operational taxonomic unit-based approaches for 16S rRNA gene sequence analysis. Appl. Environ. Microbiol. 2011;77:3219–26.
21. Mysara M, Leys N, Raes J, Monsieurs P. IPED: a highly efficient denoising tool for Illumina MiSeq Paired-end 16S rRNA gene amplicon sequencing data. BMC Bioinformatics. 2016;17:192.
22. Mysara M, Saeys Y, Leys N, Raes J, Monsieurs P. CATCh, an ensemble classifier for chimera detection in 16S rRNA sequencing studies. Appl. Environ. Microbiol. 2015;81:1573–84.
32. Hildebrand F, Tadeo R, Voigt A, Bork P, Raes J. LotuS: an efficient and user-friendly OTU processing pipeline. Microbiome. BioMed Central Ltd; 2014;2:30.
