# Supplementary material for: From reads to operational taxonomic units: an ensemble processing pipeline for MiSeq amplicon sequencing data
Source: Gigascience. 2017 Jan 18;6(2):1–10. doi: 10.1093/gigascience/giw017 (PMC5466709; doi:10.1093/gigascience/giw017)
Supplement: Reviewer_2_Report_(Original_Submission).pdf [file giw017_Reviewer_2_Report_(Original_Submission).pdf]

Reviewer Report

Title: "From Reads to Operational Taxonomic Units: an ensemble processing pipeline for MiSeq amplicon sequencing data."

Version: Original Submission **Date:** 8/24/2016

Reviewer name: Janet Williams

Reviewer Comments to Author:

Title: From Reads to Operational Taxonomic Units: an ensemble processing pipeline for MiSeq amplicon sequencing data

Reviewer: Janet Williams

Overview: Mysara et al. compare 4 different pipelines based on reads throughput, error rate and OTU accuracy. They also introduce a novel pipeline called OCToPUS (Optimized CATCh, mother, IPED, UPARSE, and SPAdes) as an alternative to these pipelines. Their approach is to incorporate beneficial aspects of various tools into a novel pipeline which will then provide an end result which is superior to any of the previous tools / pipelines used alone.

The text of the paper is very readable and well written in terms of language and flow for the paper.

As the authors state this is a "fast-evolving discipline". As such, there are recently released software and pipelines that address some of the issues and challenges that the authors address in this paper.

Would like to have seen a discussion by the authors as to why they chose the particular parts from the other tools and algorithms, i.e. what were the presumed benefits gained from implementing those approaches in their new pipeline. And did the results support their reasons for choosing those particular parts from the different pipelines?

Also, the authors state the work "rather treats the entire pipeline as a black box and assesses the accuracy using a unified evaluation process…" But it seems then that deactivation of various commands in some of the other pipelines that were being compared might influence that end result and therefore be an unfair comparison. If indeed the authors wanted to compare results at the end of each pipeline, I

would have preferred seeing a comparison of the results based on the default settings and final outcome from the various pipelines.

Level of interest:

This is an article of interest to a field that is still working on developing appropriate and accurate analysis and pipelines for microbial sequence data.

Quality of written English

Acceptable; see minor editing comments

Minor editing comments:

Table 2

Sample ID - 'v4.v5.1' should be v4.v5.I1

Line 43 - high "through-put" should be high-throughput to keep consistent with rest of text

Line 52 - need space between "IonTorrent"

Line 83 - double commas

Line 95 - sentence doesn't make sense; remove "be"

Line 96 - term should be plural

References

Line 338: Ion Torrent should be capitalized

Line 341: Illumina needs first letter capitalized

Line 352-354: Title is in title case as compared to other references; just needs consistency among references

Line 357: The "a" should be capitalized

Line 364-365: Reference in title case

Line 370: Missing page numbers for article

Line 411: Reference in title case

Line 400 and Line 442 - Quince reference is duplicated

Line 448 - Illumina should be capitalized

Line 387 and Line 471 - Edgar reference is duplicated

24. Edgar RC. Search and clustering orders of magnitude faster than BLAST. Bioinformatics. 2010;26:2460-1.

62. Edgar RC. Search and clustering orders of magnitude faster than BLAST. Bioinformatics. 2010;26:2460-1.

Line 484 - should Table 1 legend be in a different font?

Line 489 - should Table 2 legend be in a different font?

Line 505-507 - in a different font

Figure 1 - "Mothur" is capitalized

Figure 2 - "Mothur" is capitalized; OCTOPUS is all caps

Figure 3 - "Mothur" is capitalized

Figure 4 - "Mothur" is capitalized

Methods

Are the methods appropriate to the aims of the study, are they well described, and are necessary controls included? Yes

Conclusions

Are the conclusions adequately supported by the data shown? Yes

Reporting Standards

Does the manuscript adhere to the journal's guidelines on minimum standards of reporting? Yes

Statistics

Are you able to assess all statistics in the manuscript, including the appropriateness of statistical tests used? There are no statistics in the manuscript.

Quality of Written English

Please indicate the quality of language in the manuscript: Acceptable

Declaration of Competing Interests

Please complete a declaration of competing interests, considering the following questions:

- Have you in the past five years received reimbursements, fees, funding, or salary from an organisation that may in any way gain or lose financially from the publication of this manuscript, either now or in the future?
- Do you hold any stocks or shares in an organisation that may in any way gain or lose financially from the publication of this manuscript, either now or in the future?
- Do you hold or are you currently applying for any patents relating to the content of the manuscript?
- Have you received reimbursements, fees, funding, or salary from an organization that holds or has applied for patents relating to the content of the manuscript?
- Do you have any other financial competing interests?
- Do you have any non-financial competing interests in relation to this paper?

If you can answer no to all of the above, write 'I declare that I have no competing interests' below. If your reply is yes to any, please give details below.

I declare that I have no competing interests

I agree to the open peer review policy of the journal. I understand that my name will be included on my report to the authors and, if the manuscript is accepted for publication, my named report including any attachments I upload will be posted on the website along with the authors' responses. I agree for my report to be made available under an Open Access Creative Commons CC-BY license (http://creativecommons.org/licenses/by/4.0/). I understand that any comments which I do not wish to be included in my named report can be included as confidential comments to the editors, which will not be published.

I agree to the open peer review policy of the journal

To further support our reviewers, we have joined with Publons, where you can gain additional credit to further highlight your hard work (see: https://publons.com/journal/530/gigascience). On publication of this paper, your review will be automatically added to Publons, you can then choose whether or not to claim your Publons credit. I understand this statement.

Yes
