# Supplementary material for: From reads to operational taxonomic units: an ensemble processing pipeline for MiSeq amplicon sequencing data
Source: Gigascience. 2017 Jan 18;6(2):1–10. doi: 10.1093/gigascience/giw017 (PMC5466709; doi:10.1093/gigascience/giw017)
Supplement: Reviewer_2_Report_(Revision_1).pdf [file giw017_Reviewer_2_Report_(Revision_1).pdf]

Reviewer Report

Title: "From Reads to Operational Taxonomic Units: an ensemble processing pipeline for MiSeq amplicon sequencing data."

Version: Revision 1 **Date:** 10/10/2016

Reviewer name: Janet Williams

Reviewer Comments to Author:

Review of revised OCToPUS manuscript 9-29-16

Thank you for addressing the points from the previous review and making the edits in this revised manuscript.

I have two additional comments and a few more minor edits.

Line 255-261 - The authors state "the amount of reads retained by each of the workflows was dramatically differing between each of them." However, the percentages on average for 4 of them were very similar (23%, 24%, 26%, 26%). Only one was 47%. Also, it appears no statistics were actually conducted to determine if there were statistical differences among the different pipelines. So I would caution use of the words "dramatically differing."

Line 290 - "amount of reads removed by USEARCH - the pipeline with the second best performance - is drastically lower compared with other pipelines…" - Doesn't USEARCH actually reject the greatest number of reads? Or am I reading this sentence incorrectly?

Minor edits:

Line 119 - "contains of 21 species" ; perhaps the "of" should be deleted so it reads "contains 21 species"

Line 172 - reference for (Erik Aronesty, 2011) missing in references?

Line 173 - missing period following "al." to be consistent with other formatting

Line 256 - perhaps would read better as "dramatically differed" instead of "was dramatically differing"?

Line 259 - similar to above, perhaps "differs dramatically" instead of "is differing dramatically"?

Line 338 - has period at end whereas, none of the others do

Methods

Are the methods appropriate to the aims of the study, are they well described, and are necessary controls included? Yes

Conclusions

Are the conclusions adequately supported by the data shown? Yes

Reporting Standards

Does the manuscript adhere to the journal's guidelines on minimum standards of reporting? Yes

Statistics

Are you able to assess all statistics in the manuscript, including the appropriateness of statistical tests used? There are no statistics in the manuscript.

Quality of Written English

Please indicate the quality of language in the manuscript: Acceptable

Declaration of Competing Interests

Please complete a declaration of competing interests, considering the following questions:

- Have you in the past five years received reimbursements, fees, funding, or salary from an organisation that may in any way gain or lose financially from the publication of this manuscript, either now or in the future?
- Do you hold any stocks or shares in an organisation that may in any way gain or lose financially from the publication of this manuscript, either now or in the future?
- Do you hold or are you currently applying for any patents relating to the content of the manuscript?

- Have you received reimbursements, fees, funding, or salary from an organization that holds or has applied for patents relating to the content of the manuscript?
- Do you have any other financial competing interests?
- Do you have any non-financial competing interests in relation to this paper?

If you can answer no to all of the above, write 'I declare that I have no competing interests' below. If your reply is yes to any, please give details below.

I declare that I have no competing interests

I agree to the open peer review policy of the journal. I understand that my name will be included on my report to the authors and, if the manuscript is accepted for publication, my named report including any attachments I upload will be posted on the website along with the authors' responses. I agree for my report to be made available under an Open Access Creative Commons CC-BY license (http://creativecommons.org/licenses/by/4.0/). I understand that any comments which I do not wish to be included in my named report can be included as confidential comments to the editors, which will not be published.

I agree to the open peer review policy of the journal

To further support our reviewers, we have joined with Publons, where you can gain additional credit to further highlight your hard work (see: https://publons.com/journal/530/gigascience). On publication of this paper, your review will be automatically added to Publons, you can then choose whether or not to claim your Publons credit. I understand this statement.

Yes
